# Travelling Waves in a PDE–ODE Coupled Model of Cellulolytic Biofilms with Nonlinear Diffusion

**DOI:** 10.1007/s10884-022-10240-4

**Published:** 2023-01-18

**Authors:** K. Mitra, J. M. Hughes, S. Sonner, H. J. Eberl, J. D. Dockery

**Affiliations:** 1 Faculty of Science, Hasselt University, Hasselt, Belgium; 2 Department of Mathematics, University of British Columbia, Vancouver, Canada; 3 Faculty of Science, Radboud University, Nijmegen, The Netherlands; 4 Department of Mathematics and Statistics, University of Guelph, Guelph, Canada; 5 Department of Mathematical Sciences, Montana State University, Bozeman, USA

**Keywords:** Travelling waves, Stability, Degenerate diffusion, PDE–ODE system, Biofilm, 35C07, 35K65, 35Q92, 35B35, 34B08

## Abstract

We analyze travelling wave (TW) solutions for nonlinear systems consisting of an ODE coupled to a degenerate PDE with a diffusion coefficient that vanishes as the solution tends to zero and blows up as it approaches its maximum value. Stable TW solutions for such systems have previously been observed numerically as well as in biological experiments on the growth of cellulolytic biofilms. In this work, we provide an analytical justification for these observations and prove existence and stability results for TW solutions of such models. Using the TW ansatz and a first integral, the system is reduced to an autonomous dynamical system with two unknowns. Analysing the system in the corresponding phase–plane, the existence of a unique TW is shown, which possesses a sharp front and a diffusive tail, and is moving with a constant speed. The linear stability of the TW in two space dimensions is proven under suitable assumptions on the initial data. Finally, numerical simulations are presented that affirm the theoretical predictions on the existence, stability, and parametric dependence of the travelling waves.

## Introduction

In this paper we investigate travelling wave (TW) solutions for nonlinear degenerate coupled PDE–ODE systems that model the growth of cellulolytic biofilms [10]. Cellulolytic biofilms play an important role in the production of cellulosic ethanol, a renewable biofuel that can be implemented in the existing transportation infrastructure. In contrast to more traditional biofilms that form on mostly abiotic surfaces and develop colonies that grow into the surrounding aqueous phase, many cellulolytic biofilms consume and degrade the biological material that supports them and form crater like depressions, a phenomenon known as inverted colony formation. Since the nutrients are immobile, whereas, the biofilm expands spatially, a PDE–ODE model was proposed for cellulolytic biofilms in [10]. This cellulolytic biofilm model is the motivation for our study and a particular case of the following class of PDE-ODE coupled systems we consider:

1.1a $$\begin{aligned}&\partial _t M=\partial _x [D(M)\,\partial _x M] + \left( f(S)-\lambda \right) M, \end{aligned}$$

1.1b $$\begin{aligned}&\partial _t S= -\gamma \,f(S)\,M, \end{aligned}$$

where $x\in {\mathbb {R}}$ and $t>0$ represent the space and time coordinates respectively. In the model, *M* represents the biomass density, and *S* the immobilized nutrient concentration. Both are normalized, *S* taking values in the interval $[0,\infty )$ and *M* in [0, 1). Since we are interested in a TW solution of the above system, for *M* we consider the following boundary conditions at infinity,

1.2a $$\begin{aligned} M(\pm \infty ,t)=0, \quad (D(M)\,\partial _x M)(\pm \infty ,t)=0. \end{aligned}$$

For *M* and *S*, initial conditions consistent with the above are chosen. In particular, we assume that

1.2b $$\begin{aligned} {\left\{ \begin{array}{ll} M(x,0)\in [0,1), \quad S(x,0)\in [0,\infty ) &{}\text { for all } x< 0,\\ M(x,0)=0, \quad S(x,0)=1 &{}\text { for all } x\ge 0. \end{array}\right. } \end{aligned}$$

The growth and decay characteristics of the system (1.1) are represented by the constants $\lambda ,\,\gamma \in (0,1)$. For the diffusion coefficient $D:[0,1)\rightarrow [0,\infty )$ and reaction term $f:[0,\infty )\rightarrow [0,1]$ we assume the following properties:

(P1): The diffusion coefficient $D:[0,1)\rightarrow [0,\infty )$ is an increasing function in $C^1([0,1))$ which satisfies for constants $a,\,b>1$
  $$\begin{aligned} \lim \limits _{m\searrow 0} \frac{D(m)}{m^a}\in (0,\infty ),\;\;\text { and }\;\; \lim \limits _{m\nearrow 1} (1-m)^b\,D(m)\in (0,\infty ). \end{aligned}$$
(P2): The source function $f: [0,\infty )\rightarrow [0,1]$ is a strictly increasing function in $C^1([0,\infty ))$ which satisfies for a constant $\kappa \in (0,1]$,
  $$\begin{aligned} f(0)=0,\quad f'(0)=\frac{1}{\kappa },\;\;\text { and }\;\; f(1)\in (\lambda ,1]. \end{aligned}$$

In the context of cellulolytic biofilms, the production of biomass is caused by the bacteria consuming nutrients and degrading the biological material. This is modelled by the Monod reaction function *f*, whereas, the spatial spreading of biomass is modelled by a density-dependent diffusion coefficient *D*. Their corresponding expressions [10] are

1.3a $$\begin{aligned} D(m)&=\frac{\delta \,m^a}{(1-m)^b},{} & {} \delta>0, \ a,\,b>1, \end{aligned}$$

1.3b $$\begin{aligned} f(s)&= \frac{s}{\kappa +s},{} & {} 0<\kappa \ll 1. \end{aligned}$$

The porous medium type degeneracy $m^a$ in the diffusion coefficient ensures a sharp interface between the biofilm, represented by the region $\{M(x,t)>0\}$, and the surrounding region $\{M(x,t)=0\}$. The singularity $(1-M)^b$ ensures that the biomass density remains bounded by 1, see [16] and the references therein.

Cellulolytic biofilm formation has been studied both numerically and experimentally. In [30], an agent based stochastic discrete cellular automaton model was used to study the system. The model in [10], on the other hand, is the deterministic continuum model (1.1). Extensions of this model that account for attachment of cells from the aqueous phase to the biofilm were presented in [16, 23] using either Itô stochastic differential equations or random differential equations. Numerical simulations of the original deterministic model in [10] suggest the existence of TW solutions, which describe a constant rate of degradation of the cellulosic material that is utilised by the bacteria. Additional numerical evidence for this is given in [16] where a different time integration method is used, along with an independent implementation. Celluloytic biofilm systems are very difficult to observe experimentally with time-lapse microscopy techniques. Nevertheless, in [30] experiments were reported that suggest degradation of paper chads by cellulosic biofilms at a constant speed, which gives indirect evidence for TW like degradation in the biological system. Furthermore, experimental observations in [30] and in [8] indicate that the width of the microbially active band remains constant as the wave of microbial crater formation spreads. A rigorous proof of the existence and features of TW solutions of the model in [10], or an answer to the question under which conditions on parameters such TWs can be found, have so far been open problems. In this study we provide the answers to these questions. For this purpose, the initial and boundary conditions (1.2) are chosen to be consistent with the physical setting of the numerical experiments in [10].

We emphasize that, although our study is motivated and prompted by the cellulosic biofilm system, our results are valid for the significantly wider class of problems (1.1). Several examples of semilinear evolution equations coupled to an ODE through the source–term can be found in [17] as models of reactive transport through the subsurface. In mathematical biology, examples of similar systems are found in [22, Chapter 13], as well as a discussion on their TW solutions. PDE–ODE coupled systems of various type with nonlinear diffusion coefficients are used to model a variety of other physical or biological processes ranging from hysteretic flow through porous media [18], to tumor growth [13] and wound healing [14].

Standard approaches to prove the existence and stability of TW solutions for equations such as the Fisher-KPP equation do not generalize to the system (1.1). Difficulties arise through the degeneracy and singularity of the diffusion coefficient, as well as the nonlinear coupling between the PDE and the ODE, which leads to a non-monotone profile for *M*. In this paper, we use ordering of orbits in the (*M*, *S*) phase–plane to prove the existence and uniqueness of a TW solution for the system (1.1). We further derive stability results using asymptotic expansions. Moreover, we present numerical simulations that affirm the theoretical predictions on the existence, stability and parametric dependence of the TWs.

The existence of TWs for the (scalar) porous medium equation with a nonlinear source term was investigated in [4]. It was shown that under certain conditions on the coefficients, there exists a minimum speed for which TW solutions exist. The results were extended to include nonlinear advection terms in [2]. Furthermore, in [1] the stability of such TW solutions was shown in one space dimension. For the porous medium equation with Fisher-type reaction term, the existence of TWs was shown in [24], again for wave-speeds larger than a minimum value. In [3], further qualitative properties of the TW solutions were analysed. These results were generalized in [11] for equations with the biofilm diffusion coefficient *D* in (1.3a). However, the aforementioned results are limited to scalar equations with Fisher-type nonlinear reaction terms, and thus, exclude the complex interplay between the ODE and the PDE solutions. While the TW profile is a monotone function with respect to *x* in all the mentioned results, this is not the case for the TW profile of *M* in our system.

TWs for PDE–ODE coupled systems have been studied for multiphase flow through porous media in [19, 21, 27], where non-monotone profiles of *M* have been observed. The ordering of orbits in the phase–plane is also used in these papers to predict the behaviour of the TWs. The existence of TWs in two-dimensions for a PDE–ODE model was investigated in [20] in the context of hysteretic flow through porous media, and non-planar TWs were shown to exist. However, TWs in these cases originate from the advection term, rather than the source term. Non-monotone profiles have also been observed for the TWs of PDE–ODE systems arising in biology, see [13, 14] for examples. TWs for a PDE–PDE coupled model of bacteria spreading in an aqueous phase were analysed in [25]. Nevertheless, these systems differ fundamentally in their structure from (1.1). In our setting, as will be observed later, the TWs have distinctive pulse-like features which distinguish them from the examples above. They inherit a sharp front and a minimum speed of propagation like TWs of the porous medium equation. However, due to the coupling with the ODE, they exhibit a non-monotone profile with a diffusive tail.

The outline of our paper is as follows: In Sect. 2, we state the assumptions on the associated functions, and using the TW ansatz, the system (1.1) is reduced to a dynamical system with two unknowns. The existence result for the TWs is also stated, see Theorem 2.1. In Sect. 3, using phase–plane analysis we develop the auxiliary results which are then used to prove Theorem 2.1. Section 4 is dedicated to proving a linear stability result for the TWs in two space dimensions using asymptotic expansion. In Sect. 5, numerical results are presented for a discretization of the full PDE–ODE system, an ODE approach inspired by the TW analysis, and a numerical continuation approach. All three different approaches concur about the existence/non-existence of TWs in a parametric regime indicated by our theory. Furthermore, numerical results showing the influence of the parameters, and the stability of the TWs are presented. In Sect. 6, we interpret the analytical and numerical results in the context of cellulolytic biofilms, and discuss possible generalizations and future applications.

## Preliminaries and Main Result

Our aim is to investigate the existence of TW solutions for the system (1.1) consistent with the boundary and initial conditions (1.2). In this section, we reduce this system by using a TW ansatz that leads to an autonomous ODE system with three unknowns. Subsequently, we use a first integral to simplify the system further by eliminating one of the unknowns. Finally, we present our main result which states that a unique TW exists of (1.1) under some parametric conditions.

### Assumption 2.1

(TW ansatz) For a wave-speed $v>0$, and the travelling wave coordinate $\xi =x-v t$, there exist $M,S:{\mathbb {R}}\times [0,\infty )\rightarrow [0,1]$ which satisfy (1.1)–(1.2) and

$$\begin{aligned} M(x,t)=M(\xi ), \quad S(x,t)=S(\xi ). \end{aligned}$$

With this ansatz, the system (1.1) is written as

2.1a $$\begin{aligned} -&v \tfrac{\textrm{d}}{\textrm{d}\xi }M=\tfrac{\textrm{d}}{\textrm{d}\xi }\left[ D(M)\,\tfrac{\textrm{d}}{\textrm{d}\xi }M \right] + \left( f(S)-\lambda \right) M, \end{aligned}$$

2.1b $$\begin{aligned}&v\tfrac{\textrm{d}}{\textrm{d}\xi }S= \gamma \, f(S)\, M. \end{aligned}$$

The initial and boundary conditions (1.2) are transformed into

2.2a $$\begin{aligned}&M(-\infty )= (D(M)\,\tfrac{\textrm{d}}{\textrm{d}\xi }M)(- \infty )=0, \end{aligned}$$

2.2b $$\begin{aligned}&M(\xi )=0, \; S(\xi )=1\; \text { for all } \xi \ge 0. \end{aligned}$$

Additionally, we demand flux continuity at $\xi =0$, implying

2.2c $$\begin{aligned} \lim \limits _{\xi \nearrow 0} (D(M)\,\tfrac{\textrm{d}}{\textrm{d}\xi }M)(\xi )=\lim \limits _{\xi \searrow 0} (D(M)\,\tfrac{\textrm{d}}{\textrm{d}\xi }M)(\xi )=0. \end{aligned}$$

### Auxiliary Quantities and Reductions

For a given solution (*M*, *S*) of (2.1)–(2.2), the accumulated biomass $\omega :{\mathbb {R}}\rightarrow (0,\infty )$ until $\xi \in {\mathbb {R}}$, is defined as

2.3 $$\begin{aligned} \omega (\xi ):=\int _{-\infty }^\xi M,\quad \text { implying }\quad \tfrac{\textrm{d}}{\textrm{d}\xi }\omega =M \text { and } \omega (-\infty )=0. \end{aligned}$$

Using (2.1b) and (2.3), we rewrite (2.1a) as

$$\begin{aligned} -v \tfrac{\textrm{d}}{\textrm{d}\xi }M=\tfrac{\textrm{d}}{\textrm{d}\xi }\left[ D(M)\,\tfrac{\textrm{d}}{\textrm{d}\xi }M \right] + \tfrac{\textrm{d}}{\textrm{d}\xi }\left( \tfrac{v}{\gamma }S -\lambda \omega \right) . \end{aligned}$$

Integrating the above equation from $-\infty$ to $\xi$, we have using () that

$$\begin{aligned} -v M = D(M)\,\tfrac{\textrm{d}}{\textrm{d}\xi }M + \tfrac{v}{\gamma }(S-S(-\infty )) -\lambda \omega . \end{aligned}$$

Observe that *S* is a non-decreasing function by (2.1b) with $S(0)=1$, and $S(\xi )\ge 0$ for all $\xi \in {\mathbb {R}}$. Therefore, $S(-\infty )\ge 0$ is well-defined. Upon rearranging the above equation one has

2.4 $$\begin{aligned} D(M)\,\tfrac{\textrm{d}}{\textrm{d}\xi }M =\lambda \omega -v \,(M + \tfrac{1}{\gamma }(S-S(-\infty ))). \end{aligned}$$

Passing $\xi \rightarrow \infty$ and using () one further has

2.5 $$\begin{aligned} \omega (+\infty )=\tfrac{v}{\lambda \,\gamma }(1-S(-\infty )), \end{aligned}$$

which serves as a kind of Rankine-Hugoniot condition for the wave-speed *v*. Finally, using the relations above, (2.1) is rewritten as an autonomous dynamical system for *M*, *S* and $\omega$,

2.6a $$\begin{aligned}&\tfrac{\textrm{d}}{\textrm{d}\xi }M= \tfrac{1}{D(M)}[\lambda \omega -v \,(M + \tfrac{1}{\gamma }(S-S(-\infty )))], \end{aligned}$$

2.6b $$\begin{aligned}&\tfrac{\textrm{d}}{\textrm{d}\xi }S= \tfrac{\gamma }{v} \, f(S)\,M, \end{aligned}$$

2.6c $$\begin{aligned}&\tfrac{\textrm{d}}{\textrm{d}\xi }\omega =M. \end{aligned}$$

Observing that $S(\xi )\ge S(-\infty )\ge 0$ for all $\xi \in {\mathbb {R}}$, equation (2.6b) is rewritten using (2.6c) as

2.7 $$\begin{aligned} \tfrac{\textrm{d}}{\textrm{d}\xi }S=\tfrac{\gamma }{v}f(S)\tfrac{\textrm{d}}{\textrm{d}\xi }\omega \quad \text { or }\quad \tfrac{v}{\gamma f(S)} \tfrac{\textrm{d}}{\textrm{d}\xi }S=\tfrac{\textrm{d}}{\textrm{d}\xi }\omega . \end{aligned}$$

We introduce the function $F:(0,1]\rightarrow [0,\infty )$ as

2.8 $$\begin{aligned} F(s):=\int ^1_s \frac{\textrm{d}\varrho }{f(\varrho )}.\quad \text { It follows from (P2) that }\; F'(s)<0,\;\lim \limits _{s\searrow 0}F(s)=\infty ,\; F(1)=0. \end{aligned}$$

The limit $F(s)\rightarrow \infty$ for $s\searrow 0$ follows from (P2) since $f(s)\sim s/\kappa$ in a right neighbourhood of $s=0$, and consequently $F(s)= \int _s \frac{1}{f}\sim -\kappa \log (s)\rightarrow \infty$ as $s\searrow 0$. Integrating (2.7) from $\xi \le 0$ to $+\infty$ and using () one has $\tfrac{v}{\gamma } F(S)= \omega (+\infty )-\omega$, which upon rearranging and using (2.5) gives

2.9 $$\begin{aligned} \omega =\tfrac{v}{\gamma }\left[ \tfrac{1}{\lambda }(1-S(-\infty ))- F(S)\right] . \end{aligned}$$

Passing $\xi \rightarrow -\infty$ in the above equation, using $\omega (-\infty )=0$ from (2.3) and cancelling equal terms, we get

$$\begin{aligned} S(-\infty )+\lambda F(S(-\infty ))=1. \end{aligned}$$

Since *F* is strictly decreasing and convex, as evident from (P2), there can at most be two solutions of the equation $g(s)=s+ \lambda F(s)=1$ in (0, 1]. One trivial solution is $s=1$. Since $g(0)=\infty$ from (2.8), the existence of the second solution is guaranteed if $g'(1)>0$, or $F'(1)=-1/f(1)>-1/\lambda$ which holds due to (P2). Hence, we define $s_{-\infty } \in (0,1)$ as the nontrivial solution of

2.10 $$\begin{aligned} g(s_{-\infty })=s_{-\infty }+\lambda F(s_{-\infty })=1. \end{aligned}$$

#### Remark 2.1

(The value of $s_{-\infty }\in (0,1)$) Let *f* be given by the expression in (1.3). Then, $F(s)=1-s-\kappa \log (s)$. Thus, for $\kappa \ll (1-\lambda )/\lambda$, one has

$$\begin{aligned} s_{-\infty }\approx \exp \left( -\tfrac{1-\lambda }{\kappa \lambda }\right) . \end{aligned}$$

For the parameters $\lambda =0.42$, $\gamma =0.4$ and $\kappa =0.01$ used in [10], we estimate that

$$\begin{aligned} s_{-\infty }\approx 10^{-60}, \end{aligned}$$

which is negligible for all practical purposes. This suggests that in this parameter regime, the substrate is fully depleted after the TW has passed. In Sect. 5.3, we provide an example of parameters for which $s_{-\infty }\approx 0.11$, and hence, there remains a significant level of residual substrates.

Finally, substituting (2.9) into (2.6) we get the reduced autonomous dynamical system with only two unknowns *M* and *S*, i.e.,

2.11a $$\begin{aligned}&\tfrac{\textrm{d}}{\textrm{d}\xi }M= \tfrac{v}{\gamma \, D(M)}[(1-S-\lambda F(S)) -\gamma M], \end{aligned}$$

2.11b $$\begin{aligned}&\tfrac{\textrm{d}}{\textrm{d}\xi }S= \tfrac{\gamma }{v}\, f(S)\,M. \end{aligned}$$

The revised boundary conditions for this system are

2.12 $$\begin{aligned} M(-\infty )=0,\; S(-\infty )=s_{-\infty } \text { and } M(\xi )=0,\; S(\xi )=1 \text { for all }\xi \ge 0. \end{aligned}$$

This will be the main system analysed in this paper.

#### Remark 2.2

(The flux conditions at $\xi =0$ and $\xi =-\infty$) Observe that any solution (*M*, *S*) of the system ()–(2.12), automatically satisfies the boundary condition for the flux, i.e., $D(M)\tfrac{\textrm{d}}{\textrm{d}\xi }M=0$ at $\xi =0$ and $\xi =-\infty$.

### Main Theorem

For the rest of this study, we focus on the following parametric regime: for *F* defined in (2.8), and $g=\mathbb {I}+\lambda F$, let $\lambda ,\gamma \in (0,1)$ be such that

2.13 $$\begin{aligned} g(f^{-1}(\lambda ))= f^{-1}(\lambda ) + \lambda F(f^{-1}(\lambda ))\le 1-\gamma . \end{aligned}$$

Observe that, the function *g*(*y*) takes its minimum value $g_{\min }$ at $y=f^{-1}(\lambda )$. The condition (P2) then guarantees that $f^{-1}(\lambda )\in (0,1)$. The existence of $s_{-\infty }\in (0,1)$ in (2.10) proves that $g_{\min }<1$. Assumption (2.13) enforces a stronger condition, i.e., that $g_{\min }\le 1-\gamma$. For the Monod reaction function $f(s)=s/(\kappa + s)$, for which one has $f^{-1}(\lambda )=\kappa \lambda /(1-\lambda )$ and $F(s)=1-s-\kappa \log (s)$, the condition can be stated in an explicit form, i.e.,

2.14 $$\begin{aligned} 0<\gamma + \lambda + \kappa \lambda \,(1-\log (\kappa \lambda /(1-\lambda )))\le 1. \end{aligned}$$

We introduce the following important integral:

2.15a $$\begin{aligned} \mathcal {G}(s)&:=\int _s^1 (\varrho +\lambda F(\varrho )-(1-\gamma ))\, \frac{\textrm{d}\varrho }{f(\varrho )} \end{aligned}$$

2.15b $$\begin{aligned}&\overset{(2.8)}{=}\int _s^1\frac{\varrho }{f(\varrho )}\,\textrm{d}\varrho +\frac{\lambda }{2}\, F^2(s)-(1-\gamma )\,F(s). \end{aligned}$$

**Fig. 1:**
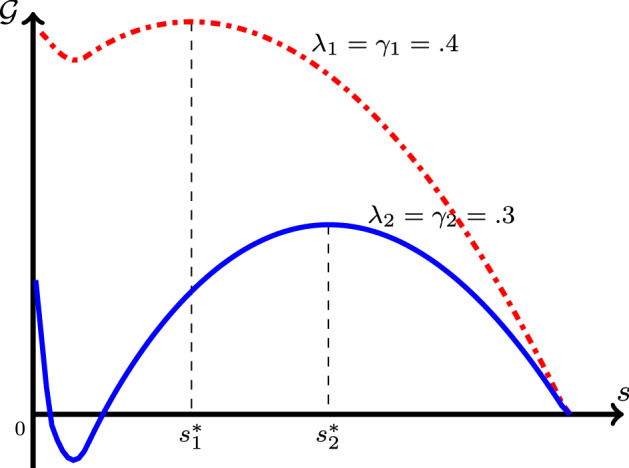
The plot of $\mathcal {G}$ as defined in () for $f(s)=s/(\kappa +s)$, $\lambda _1=\gamma _1=0.4$ and $\lambda _2=\gamma _2=0.3$. The points $s=s^{*}\in (0,1)$ marked are the solutions of $s+ \lambda F(s)=1-\gamma$ (Color figure online)

This representation follows using $F'=-1/f$ and $F(1)=0$ from (2.8). The shape of the $\mathcal {G}$-integral is shown in Fig. 1. Observe that, $\mathcal {G}(1)=0$, $\mathcal {G}'(1)=-\gamma /f(1)<0$ (since $F(1)=0$), and $\mathcal {G}(s)$ remains a decreasing function for all $s\in (s^*,1)$, where $s^*\in (0,1)$ (formally introduced in (3.6)) solves $s^*+ \lambda F(s^*)=1-\gamma$, i.e., $\mathcal {G}'(s^*)=0$. Hence, $\mathcal {G}> 0$ in an interval $(s_g,1)$ where $s_g\in (0, s^*)$. Also from (2.15b), $\mathcal {G}(s)\rightarrow +\infty$ as $s\searrow 0$ since $F(s)\rightarrow \infty$ in this case, see (2.8). Hence, depending on the parameter values, $\mathcal {G}$ might or might not have a negative part. This has a profound effect on the existence of TWs as stated in our main theorem below.

#### Theorem 2.1

(Existence of the TW solution) Assume (P1)–(P2). Let (2.13) be satisfied and let $\mathcal {G}(s)>0$ for all $s\in (s_{-\infty },1)$. Then there exists a unique $v>0$ such that a travelling wave solution $(M,S):{\mathbb {R}}\rightarrow [0,1]^2$ with $D(M)\tfrac{\textrm{d}}{\textrm{d}\xi }M\in C({\mathbb {R}})$ and $S\in C^1({\mathbb {R}})$ exists satisfying ()–(2.12).

#### Remark 2.3

(Conditions on existence) The condition (2.13), used in Theorem 2.1, provides upper bounds for $\lambda$ and $\gamma$, whereas, the condition $\mathcal {G}(s)>0$ for $s\in (s_{-\infty },1)$ provides lower bounds for $\lambda$ and $\gamma$ for the existence of the TW solutions. We show in Proposition 3.2 that the latter condition is also a necessary condition. Condition (2.13) is however only a sufficient condition as a numerical example is given in Sect. 5.2 that indicates that a TW solution exists despite the fact that (2.13) is violated. Nevertheless, this technical assumption yields a neat geometric argument of why non-monotone TWs will exists for the system as elaborated in Sect. 3.1. Without this condition, our approach does not allow us to prove the existence of a non-monotone TW.

## The Existence of Travelling Waves

In this section we prove Theorem 2.1 by analysing the dynamical system (). For this purpose, we apply a suitable coordinate transformation that reduces the system to a form that is analysed in the phase–plane. For a fixed $v>0$, we show in Sects. 3.2 and 3.3 that an orbit, corresponding to a sharp front, exists and is unique. In Sect. 3.4 we show that these orbits are ordered with respect to *v*. The ordering is used to prove the existence of TW solutions as well as to show that the condition $\mathcal {G}>0$ in Theorem 2.1 is a necessary condition.

### The Phase–Plane

For a given orbit $\xi \mapsto (M,S)$ satisfying (), the scaled TW coordinate $\tau$ is defined by the coordinate transform

3.1 $$\begin{aligned} \tau (\xi ):=\int _{0}^\xi \tfrac{\textrm{d}\varrho }{D(M(\varrho ))},\quad \text { implying } \;\tfrac{\textrm{d}}{\textrm{d}\xi }\tau = \tfrac{1}{D(M(\xi ))}. \end{aligned}$$

Moreover, to shorten notation, we introduce

3.2 $$\begin{aligned} \ell (s;\lambda ,\gamma ):=\frac{1}{\gamma }[(1-s)-\lambda F(s)], \end{aligned}$$

which from (P2) and (2.8) has the properties

3.3a $$\begin{aligned}&\ell \in C^1((0,1]),\; \ell '(s)>0 \text { for } s<s_{\textrm{M}}:=f^{-1}(\lambda ), \text { and } \ell '(s)<0 \text { for } s>s_{\textrm{M}}; \end{aligned}$$

3.3b $$\begin{aligned}&\ell (1)=0,\text { and } \lim \limits _{s\searrow 0}\ell (s)=-\infty . \end{aligned}$$

Observe that, in terms of the function *g* introduced in Sect. 2, $\ell (s)=\gamma ^{-1}[1-g(s)]$ (recall that $g=\mathbb {I}+\lambda \,F$). In the following sections, we will only use the properties () of $\ell$, and will not further use *F* or *g*, to keep the notation as clear as possible. From (2.15a), we additionally have that

3.4 $$\begin{aligned} \mathcal {G}(s)=\gamma \int _s^1 \left( \frac{1-\ell (\varrho )}{f(\varrho )}\right) \textrm{d}\varrho . \end{aligned}$$

With the coordinate transform (3.1) and the definition (3.2), system () is re-written as

3.5a $$\begin{aligned}&\tfrac{\textrm{d}}{\textrm{d}\tau }M= v\,[\ell (S)- M], \end{aligned}$$

3.5b $$\begin{aligned}&\tfrac{\textrm{d}}{\textrm{d}\tau }S= \tfrac{\gamma }{v}\,f(S)\, M D(M). \end{aligned}$$

**Fig. 2:**
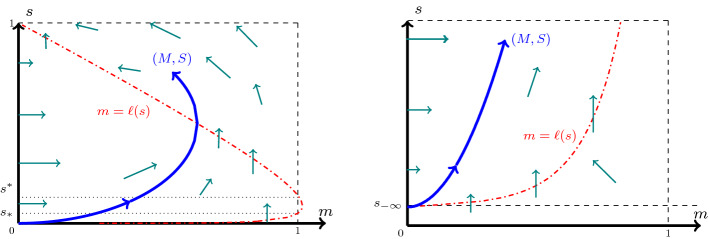
(left) The direction of orbits of the dynamical system () in the phase–plane $[0,1)\times (0,1]$. (right) A zoomed view into the phase–plane near the equilibrium point $(0,s_{-\infty })$. An orbit (*M*, *S*) originating from $(0,s_{-\infty })$, and the nullcline $m=\ell (s)$ are also shown (Color figure online)

Since this is an autonomous system, we look into the phase–plane $[0,1)\times (0,1]$. Henceforth, (*m*, *s*) will represent a point in this phase–plane, and (*M*, *S*) will denote the orbits in this plane. The directions of the orbits are shown in Fig. 2. The line $m=\ell (s)$ is highlighted and represents the nullcline of *M*, i.e., the points where $\tfrac{\textrm{d}}{\textrm{d}\tau }M=0$. Due to the restriction (2.13) imposed, this nullcline intersects the line $m=1$ at precisely two points $s_*$ and $s^*$ if a strict inequality holds in (2.13), and at one point if equality holds. This follows from the properties of $\ell$ in () along with the observation that

$$\begin{aligned} \ell (s_{\textrm{M}})=\gamma ^{-1}[1-s_{\textrm{M}}-\lambda F(s_{\textrm{M}})]\overset{(2.13)}{\ge }1,\quad \text { where } s_{\textrm{M}}=f^{-1}(\lambda ). \end{aligned}$$

Hence, the *s*-coordinates $s_*,\,s^*\in (0,1)$ satisfying

3.6 $$\begin{aligned} s_*:=\min \{s\in (0,1): \ell (s)=1\}\le s_{\textrm{M}},\quad s^*:=\max \{s\in (0,1): \ell (s)=1\}\ge s_{\textrm{M}}, \end{aligned}$$

exist (consistent with the definition of $s^*\in (0,1)$ below ()), with $s_*=s^*=s_{\textrm{M}}$ if equality holds in (2.13). Consequently, we have the following:

#### Lemma 3.1

(The existence of orbits) Let $v>0$ be given, (2.13) be satisfied and let $(M_0,S_0)\in \mathfrak {R}:=[0,1)\times [s_{-\infty },1].$ Then, there exists a unique orbit $\tau \mapsto (M,S)\in (C^1({\mathbb {R}}))^2$, satisfying () with $(M,S)(0)=(M_0,S_0)$. The equilibrium points of the system () are $(0,s_{-\infty })$ and (0, 1) with $s_{-\infty }\in (0,1)$ satisfying (2.10). If $(M_0,S_0)$ is not an equilibrium point, then

(i) for $\tau >0$, the orbit either exits $\mathfrak {R}$ through the line $\{s=1\}$ or ends at (0, 1).
(ii) for $\tau <0$, the orbit enters $\mathfrak {R}$ either though the line segment $\{m=0,s\ge s_{-\infty }\}$ or through $\{s=s_{-\infty }\}$.

To avoid distinguishing too many cases, we will assume $s_*<s^*$ in the rest of the paper. The case $s_*=s^*$ is a straightforward extension.

#### Proof

Observe that, orbits satisfying () are locally well-posed at any point in $[0,1)\times [s_{-\infty },1]$. This follows from the Picard–Lindelöf theorem since the right hand sides of () are locally–Lipschitz with respect to *M* and *S* for $M<1$ and $S\in [s_{-\infty },1]$.

*(i)* The direction of the orbits implies that (*M*, *S*) can exit $\mathfrak {R}$ through the boundaries $\{m=1\}$ or $\{s=1\}$. To rule out the line $\{m=1\}$, we assume the contrary, i.e., we suppose there exists $\tau _1>0$ such that $M(\tau _1)=1$ and $M(\tau )<1$ for all $0<\tau <\tau _1$. Then, (*M*, *S*) satisfies () for all $\tau <\tau _1$. It is straightforward to see that $S(\tau _1)\in [s_*,s^*]$ since $\lim _{\tau \nearrow \tau _1}\tfrac{\textrm{d}}{\textrm{d}\tau }M=v\,[\ell (S(\tau _1))-1]$ and the definition of $\tau _1$ demands that $\lim _{\tau \nearrow \tau _1}\tfrac{\textrm{d}}{\textrm{d}\tau }M\ge 0$. Using the intermediate value theorem, for a given $\varepsilon <1-M_0$, there exists $\tau _\varepsilon \in (0,\tau _1)$ such that $M(\tau _\varepsilon )=1-\varepsilon$. Moreover, observe that there exists a constant $C>0$ such that

3.7 $$\begin{aligned} \tfrac{\textrm{d}}{\textrm{d}\tau }M\le C \text { in } (\tau _\varepsilon ,\tau _1),\;\text { or, integrating in } (\tau _\varepsilon ,\tau _1),\quad \tau _1-\tau _\varepsilon \ge \frac{\varepsilon }{C}. \end{aligned}$$

From (3.5b) and using (3.7), for some constants $C_{1/2}>0$ independent of $\varepsilon$, one has

$$\begin{aligned} S(\tau _1)-S(\tau _\varepsilon )= & {} \int _{\tau _\varepsilon }^{\tau _1} \tfrac{\gamma }{v} f(S) M D(M) \textrm{d}\tau \\\ge & {} C_1 \int _{\tau _\varepsilon }^{\tau _1} D(1-\varepsilon )\,\textrm{d}\tau \overset{\mathrm{(P1)}}{\ge }C_2\frac{\tau _1-\tau _\varepsilon }{\varepsilon ^b}\overset{(3.7)}{\ge }\frac{C_2}{ C\varepsilon ^{b-1}}\rightarrow \infty , \end{aligned}$$

as $\varepsilon \rightarrow 0$ (note that $b>1$). Since $S(\tau _\varepsilon )>0$, this contradicts $S(\tau _1)\in [s_*,s^*]$, thus proving that (*M*, *S*) cannot exit through $\{m=1\}$.

*(ii)* For $\tau <0$, the fact that the orbits can enter through the mentioned segments is clear. The orbit cannot enter through the boundary $\{s=1\}$ since *S* is strictly increasing in $\mathfrak {R}$ for $m>0$, and (0, 1) is an equilibrium point. The fact that the orbit cannot enter through $\{m=1\}$ follows similarly as the proof of point (i).

#### Remark 3.1

(The travelling wave solutions avoid the degeneracy at $m=1$) The proof above shows that, for any TW solution (*M*, *S*) satisfying ()–(2.12), there exists a constant $\varepsilon >0$ such that

$$\begin{aligned} 0\le M(\xi )\le 1-\varepsilon , \text { for all } \xi \in {\mathbb {R}}. \end{aligned}$$

Hence, the degeneracy of the diffusion coefficient *D*, due to the possibility of $D(M)\rightarrow \infty$ as $M\nearrow 1$, is avoided.

### The Orbit Connecting with (0, 1)

For any orbit (*M*, *S*) described in Lemma 3.1, *S* is strictly increasing for all $\tau \in {\mathbb {R}}$ provided $(M,S)\in \mathfrak {R}$ and $M>0$. Hence, for a given $s\in (s_{-\infty },1)$ there can exist at most one $\tau \in {\mathbb {R}}$ such that $S(\tau )=s$. This allows us to introduce the unique mapping $S\mapsto M$ through the following function.

#### Definition 3.1

(The $\mathfrak {M}$–map) For a given $(M_0,S_0)\in \mathfrak {R}$, let (*M*, *S*) be the unique orbit $\tau \mapsto (M,S)\in (C^1({\mathbb {R}}))^2$, satisfying () and $(M,S)(0)=(M_0,S_0)$. Then the continuous function $\mathfrak {M}:[s_{-\infty },1]\rightarrow [0,1)$ is defined as

3.8 $$\begin{aligned} \mathfrak {M}(s):={\left\{ \begin{array}{ll} M(\tau ) &{}\text { if there exists}\,\tau \in {\mathbb {R}}\text { such that } S(\tau )=s,\\ 0 &{}\text { otherwise}. \end{array}\right. } \end{aligned}$$

Let us introduce the function

3.9 $$\begin{aligned} \Phi (m):= \int _0^m \varrho \, D(\varrho )\,\textrm{d}\varrho , \text { such that } \Phi '(m)=m D(m)\ge 0. \end{aligned}$$

Observe that $\mathfrak {M}$ satisfies $\mathfrak {M}(S_0)=M_0$, and for all $\mathfrak {M}>0$,

3.10a $$\begin{aligned} \dfrac{\textrm{d}\mathfrak {M}}{\textrm{d}s}= \dfrac{v^2}{\gamma }\dfrac{\ell (s)-\mathfrak {M}(s)}{f(s)\, \mathfrak {M}\, D(\mathfrak {M})}. \end{aligned}$$

Using (3.9), we alternatively rewrite the equation above as

3.10b $$\begin{aligned} \dfrac{\textrm{d}\Phi (\mathfrak {M})}{\textrm{d}s}=\dfrac{v^2}{\gamma f(s)} [\ell (s)- \mathfrak {M}(s)]. \end{aligned}$$

Our focus will be on a specific group of maps $\mathfrak {M}$ which originate from $(\varepsilon ,1)$.

#### Lemma 3.2

For fixed $v,\,\varepsilon >0$ and $(M_0,S_0)=(\varepsilon ,1)$, let $\mathfrak {M}^{\varepsilon }$ denote the $\mathfrak {M}$-mapping in the sense of Definition 3.1. For $a>1$ introduced in (P1) and the function *F* in (2.8), let $\underline{\mathfrak {M}}:(0,1]\rightarrow [0,1]$ solve

$$\begin{aligned} \int _0^{\underline{\mathfrak {M}}(s)} \frac{D(\varrho )}{\varrho ^{a-1}}\, \textrm{d}\varrho = \frac{v^2}{\gamma } F(s). \end{aligned}$$

Then there exists $\underline{s}\in (0,1)$ independent of $\varepsilon$, such that

$$\begin{aligned} \ell (s)< \underline{\mathfrak {M}}(s)< \mathfrak {M}^\varepsilon (s) \text { for all } \underline{s}\le s< 1. \end{aligned}$$

#### Proof

Observe from (P1) that $\underline{\mathfrak {M}}$ is well-defined and satisfies the ODE,

3.11 $$\begin{aligned} \dfrac{\textrm{d}\Phi (\underline{\mathfrak {M}})}{\textrm{d}s}= -\frac{v^2}{\gamma } \dfrac{\underline{\mathfrak {M}}^a}{f(s)} \end{aligned}$$

and $\underline{\mathfrak {M}}(1)=0$. Subtracting (3.10b) and integrating in (*s*, 1) one gets

3.12 $$\begin{aligned} \Phi (\mathfrak {M}^\varepsilon (s))-\Phi (\underline{\mathfrak {M}}(s))=&\, \Phi (\varepsilon )+ \frac{v^2}{\gamma }\int _s^1 \left[ (\mathfrak {M}^\varepsilon (\varrho )-\underline{\mathfrak {M}}(\varrho )) + (\underline{\mathfrak {M}}(\varrho )-\underline{\mathfrak {M}}^a(\varrho ) -\ell (\varrho )) \right] \frac{\textrm{d}\varrho }{f(\varrho )} \nonumber \\ =&\, \Phi (\varepsilon )+\frac{v^2}{\gamma }\int _s^1 \frac{1}{f(\varrho )} (\mathfrak {M}^\varepsilon -\underline{\mathfrak {M}})(\varrho ) \nonumber \\&+\, \frac{v^2}{\gamma }\int _s^1 \frac{\underline{\mathfrak {M}}(\varrho )}{f(\varrho )} \left( 1-\underline{\mathfrak {M}}^{a-1}(\varrho ) -\tfrac{\ell (\varrho )}{\underline{\mathfrak {M}}(\varrho )}\right) . \end{aligned}$$

Note that, $\ell '(1)= -\gamma ^{-1}(1-\lambda /f(1))\overset{\mathrm{(P2)}}{<}0$, whereas $\underline{\mathfrak {M}}(s)\rightarrow 0$ as $s\rightarrow 1$, and therefore,

$$\begin{aligned} \frac{d\underline{\mathfrak {M}}}{ds}=-\frac{v^2}{\gamma }\frac{\underline{\mathfrak {M}}^{a-1}}{f(s)\,D(\underline{\mathfrak {M}})}\rightarrow -\infty \text { as } s\rightarrow 1. \end{aligned}$$

Hence, using L’Hôpital’s rule, there exists $\underline{s}\in (0,1)$ independent of $\varepsilon$, such that

3.13 $$\begin{aligned} \left( 1-\underline{\mathfrak {M}}^{a-1}(s) -\frac{\ell (s)}{\underline{\mathfrak {M}}(s)}\right) \ge 0 \text { for all } \underline{s}\le s<1. \end{aligned}$$

Observe that $\mathfrak {M}^\varepsilon (s)> \underline{\mathfrak {M}}(s)$ in a left neighbourhood of $s=1$, simply because $\mathfrak {M}^\varepsilon (1)=\varepsilon >0=\underline{\mathfrak {M}}(1)$. Then, (3.12)–(3.13) imply that $\mathfrak {M}^\varepsilon (s)> \underline{\mathfrak {M}}(s)$ for all $\underline{s}\le s<1$. To see this, assume the contrary, i.e., $\mathfrak {M}^\varepsilon (s_1)=\underline{\mathfrak {M}}(s_1)$ for some $s_1\in (\underline{s},1)$ and $\mathfrak {M}^\varepsilon (s)> \underline{\mathfrak {M}}(s)$ for $s_1<s<1$. Then, from (3.12)–(3.13) we have $\Phi (\mathfrak {M}^\varepsilon (s_1))>\Phi (\underline{\mathfrak {M}}(s_1))$, thus contradicting our assumption. This concludes the proof.

#### Theorem 3.1

**Fig. 3:**
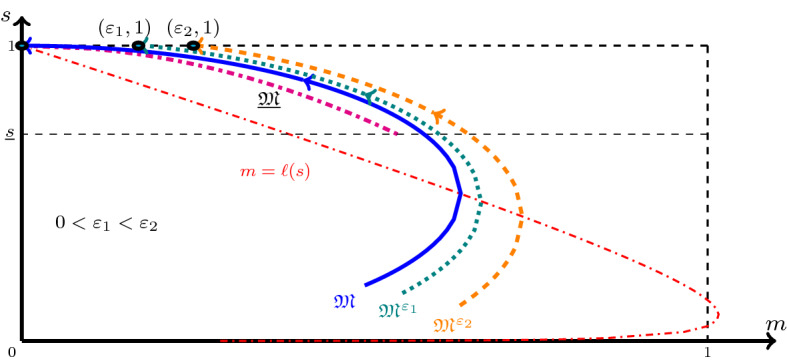
The $\mathfrak {M}$-mapping, introduced in Theorem 3.1 with $\mathfrak {M}(1)=0$, and two $\mathfrak {M}^{\varepsilon }$-mappings, introduced in Lemma 3.2 with $\mathfrak {M}^{\varepsilon }(1)=\varepsilon$ for $\varepsilon =\varepsilon _1>0$ and $\varepsilon =\varepsilon _2>\varepsilon _1$. The function $\underline{\mathfrak {M}}$ defined in Lemma 3.2, providing a lower bound, is also shown (Color figure online)

(Existence of an orbit connecting with (0, 1)) Let $v>0$ be fixed and (2.13) be satisfied. Let $\mathfrak {M}^\varepsilon$ denote the $\mathfrak {M}$-mapping introduced in Lemma 3.2 with $\mathfrak {M}^\varepsilon (1)=\varepsilon$. Then there exists a function $\mathfrak {M}:[s_{-\infty },1]\rightarrow [0,1)$ which satisfies () with $\mathfrak {M}(1)=0$ and for all $s\in [s_{-\infty },1]$, $\mathfrak {M}^\varepsilon (s)\rightarrow \mathfrak {M}(s)$ as $\varepsilon \rightarrow 0$ (see Fig. 3). Moreover, define the function $\zeta :(s_{-\infty },1]\rightarrow (-\infty ,0]$ (the $\zeta$-$\textrm{map})$ as

3.14 $$\begin{aligned} \zeta (s):=-\frac{v}{\gamma }\int _s^1 \frac{\textrm{d}\varrho }{f(\varrho )\,\mathfrak {M}(\varrho )}. \end{aligned}$$

Then $\zeta$ is differentiable and increasing with *s* whenever $\mathfrak {M}(s)>0$, and $\lim _{s\nearrow 1}\zeta (s)=0$. For any $s\in (s_{-\infty },1]$ and $\zeta (s)\in {\mathbb {R}}^-$, defining $\xi =\zeta (s)$, $M=\mathfrak {M}(s)$ and $S=s$, the mapping $\xi \mapsto (M,S)$ solves ().

#### Proof

For a fixed $v>0$ and $s\in (s_{-\infty },1]$,

3.15 $$\begin{aligned}&\mathfrak {M}_{\varepsilon _1}(s)\le \mathfrak {M}_{\varepsilon _2}(s)<1&\text { if } 0<\varepsilon _1<\varepsilon _2<1,\nonumber \\&\text {and the equality holds only if }&\mathfrak {M}_{\varepsilon _{1}}(s)= \mathfrak {M}_{\varepsilon _{2}}(s)=0. \end{aligned}$$

This is evident since orbits, corresponding to initial values $(\varepsilon _1,1)$ and $(\varepsilon _2,1)$, do not intersect in the interior of $\mathfrak {R}$ due to uniqueness of solutions, see Lemma 3.1. The lemma further yields that $\mathfrak {M}^{\varepsilon }<1$ if $\varepsilon <1$. Since, $\mathfrak {M}^\varepsilon (s)$ is bounded below by $\underline{\mathfrak {M}}(s)$ for $s\in (\underline{s},1)$, see Lemma 3.2, there exists $\mathfrak {M}(s)$ such that

3.16 $$\begin{aligned} \mathfrak {M}(s):=\lim \limits _{\varepsilon \searrow 0} \mathfrak {M}^\varepsilon (s) \;\; \text { for } s\in (s_{-\infty },1),\;\; \text { and }\;\; \mathfrak {M}(s)\ge \underline{\mathfrak {M}}(s)>0 \text { for } s\in (\underline{s},1), \end{aligned}$$

see Fig. 3. Let us take $s\in (\underline{s},1)$. Observe that by (3.10b), $\mathfrak {M}^\varepsilon$ satisfies in this interval

3.17 $$\begin{aligned} \Phi (\mathfrak {M}^\varepsilon (s))= \Phi (\varepsilon )+ \frac{v^2}{\gamma }\int _s^1 (\mathfrak {M}^\varepsilon (\varrho )-\ell (\varrho ))\,\frac{\textrm{d}\varrho }{f(\varrho )}. \end{aligned}$$

Due to (3.15), if $\varepsilon _0\in (0,1)$, then $\mathfrak {M}^\varepsilon$ is uniformly bounded away from 1 in $[s_{-\infty },1]$ for all $\varepsilon \in (0,\varepsilon _0]$. Thus, $\Phi$ can be assumed to be locally Lipschitz in the above equation. Hence, passing the limit $\varepsilon \rightarrow 0$, we get for all $s\in (\underline{s},1)$,

3.18 $$\begin{aligned} \Phi (\mathfrak {M}(s))= \frac{v^2}{\gamma }\int _s^1 (\mathfrak {M}(\varrho )-\ell (\varrho ))\,\frac{\textrm{d}\varrho }{f(\varrho )}, \end{aligned}$$

which upon differentiation proves that $\mathfrak {M}$ satisfies () in $(\underline{s},1)$ with $(M_0,S_0)=(0,1)$. For $s<\underline{s}$, the mapping $\mathfrak {M}$ is simply extended by solving the equation () with $(M_0,S_0)=(\mathfrak {M}(\underline{s}),\underline{s})\in \mathfrak {R}$. The existence of $\mathfrak {M}$ in this case also follows from the existence of orbits, i.e., Lemma 3.1.

The differentiability and monotonicity of the function $\zeta$ is obvious from (3.14). To prove that $\lim _{s\nearrow 1} \zeta (s)=0$, we estimate for any $s\in (\underline{s},1)$,

3.19 $$\begin{aligned} 0< \int _s^1 \frac{\textrm{d}\varrho }{f(\varrho )\,\mathfrak {M}(\varrho )}\overset{(3.15)}{\le }\int _s^1 \frac{\textrm{d}\varrho }{f(\varrho )\,\underline{\mathfrak {M}}(\varrho )}\overset{(3.11)}{=} \frac{\gamma }{v^2}\int ^{\underline{\mathfrak {M}}(s)}_0 \frac{D(m)}{m^a}\textrm{d}m<\infty . \end{aligned}$$

Hence, $\zeta (s)>-\infty$ for all $s\in (\underline{s},1]$, and passing to the limit $s\rightarrow 1$ one obtains that $\zeta (1^-)=0$.

Differentiating (3.14) and using (3.10a), it immediately follows that the mapping $\xi \mapsto (M,S)$ solves ().

#### Corollary 3.1

(Behaviour of the orbit connecting to (0, 1)) Let (*M*, *S*) be the orbit defined in Theorem 3.1. Then there exists $\underline{\xi }\in {\mathbb {R}}^-\cup \{-\infty \}$ such that $(M,S)\in \mathfrak {R}=[0,1)\times [s_{-\infty },1]$ for all $\xi \ge \underline{\xi }$. In $\mathfrak {R}\cap \{s\ge s_*\}$, both *M* and *S* increase with $\xi$ until $M=\ell (S)$ is satisfied for some $S>s^*$ $(s_*,\;s^*\in (s_{-\infty },1)$ defined in (3.6)), after which *M* decreases and *S* remains increasing. Finally, $(M,S)=(0,1)$ for all $\xi \ge 0$.

The statement is evident from the direction of orbits in the phase–plane, Fig. 2, Theorem 3.1 and Lemma 3.1. Below, we prove that (*M*, *S*) is the unique orbit which connects with (0, 1) at a finite $\xi$-coordinate.

### Uniqueness of the Orbit Connecting with (0, 1) at $\xi =0$

For a given $v>0$, there are in fact infinitely many orbits (*M*, *S*) that connect to (0, 1) as $\xi \rightarrow +\infty$. However, only one orbit, the one constructed in Sect. 3.2, connects to (0, 1) at $\xi =0$. This statement will be proven below. This is a common phenomenon for TW solutions of degenerate diffusion equations [3, 4] and the unique orbit corresponds to the TW with minimum speed in these cases.

#### Proposition 3.1

(Uniqueness of the orbit connecting to (0, 1) for some $\xi \in {\mathbb {R}}$) For a fixed $v>0$, let $(\widetilde{M},\widetilde{S})\in (C^1({\mathbb {R}}))^2$ be an orbit satisfying () and connecting with (0, 1) from $\{s<1\}$. Let $\widetilde{\mathfrak {M}}$ and $\widetilde{\zeta }$ denote the corresponding $\mathfrak {M}$-mapping (Definition 3.1) and $\zeta$-mapping (Theorem 3.1) of $(\widetilde{M},\widetilde{S})$ with $\widetilde{\xi }=\widetilde{\zeta }(1)$. Then, $\widetilde{\xi }<\infty$ if and only if $(\widetilde{M},\widetilde{S})$ is the orbit defined in Theorem 3.1.

#### Proof

We first show that

3.20 $$\begin{aligned} \widetilde{\xi }=\widetilde{\zeta }(1)<\infty \quad \text { implies }\quad \frac{\textrm{d}}{\textrm{d}s}\widetilde{\mathfrak {M}}(1)=-\infty . \end{aligned}$$

Assume the contrary, i.e, $\frac{\textrm{d}}{\textrm{d}s}\widetilde{\mathfrak {M}}(1)>-\infty$. Then, there exists $s\in (s_{-\infty },1)$ and $L>0$ such that

$$\begin{aligned} \widetilde{\mathfrak {M}}(\varrho )\le L\,(1-\varrho ) \text { for all } \varrho \in [s,1]. \end{aligned}$$

This implies $\widetilde{\xi }=\widetilde{\zeta }(1)=\infty$ since, writing formally,

$$\begin{aligned} \widetilde{\zeta }(1)-\widetilde{\zeta }(s)=\frac{v}{\gamma } \int ^1_s \frac{\textrm{d}\varrho }{f(\varrho )\,\widetilde{\mathfrak {M}}(\varrho )}\ge \frac{v}{\gamma } \int ^1_s \frac{\textrm{d}\varrho }{L\,f(1)\,(1-\varrho )}= \infty . \end{aligned}$$

Now, let us assume $\frac{\textrm{d}}{\textrm{d}s} \widetilde{\mathfrak {M}}(1)=-\infty$. Since $\widetilde{\mathfrak {M}}$ is continuous and differentiable in a left neighbourhood of $s=1$, and $\ell '(1)<0$ is bounded, for any given $\nu \in (0,1)$, by the L’Hôpital’s rule, there exists $\widetilde{s}_\nu \in (0,1)$ such that

3.21 $$\begin{aligned} \frac{\ell (s)}{\widetilde{\mathfrak {M}}(s)} <1-\nu \text { for all } s\in [\widetilde{s}_\nu ,1]. \end{aligned}$$

Let (*M*, *S*) be the orbit defined in Theorem 3.1 with $\mathfrak {M}$ and $\zeta$ being the corresponding mappings. To shorten notations we introduce

3.22 $$\begin{aligned} \phi =\Phi (\mathfrak {M}),\quad \widetilde{\phi }=\Phi (\widetilde{\mathfrak {M}}),\; \text { and }\; \eth \phi =\phi -\widetilde{\phi }. \end{aligned}$$

Since $\mathfrak {M}(s)=\lim _{\varepsilon \searrow 0}\mathfrak {M}^\varepsilon (s)$, see Theorem 3.1, the ordering of orbits for a fixed $v>0$ implies that

3.23 $$\begin{aligned} \mathfrak {M}(s)\ge \widetilde{\mathfrak {M}}(s),\quad \phi \ge \widetilde{\phi },\quad \eth \phi \ge 0,\; \text { for all } s\in (s_{-\infty },1). \end{aligned}$$

From (3.10b) applied to $\mathfrak {M}$ and $\widetilde{\mathfrak {M}}$, we then obtain

3.24 $$\begin{aligned} \frac{\textrm{d}(\eth \phi )}{\textrm{d}s}= -\frac{v^2}{\gamma f(s)}[\Phi ^{-1}(\phi )-\Phi ^{-1}(\widetilde{\phi })]. \end{aligned}$$

Since $\Phi$ is convex and strictly increasing, $\Phi ^{-1}$ exists and is concave, and $\{\Phi ^{-1}\}'(\Phi (m))=1/\Phi '(m)=1/(m\,D(m))$. Hence, (3.23) implies $\Phi ^{-1}(\phi )-\Phi ^{-1}(\widetilde{\phi })\le \{\Phi ^{-1}\}'(\widetilde{\phi })\, \eth \phi ={\eth \phi }/{(\widetilde{\mathfrak {M}}\,D(\widetilde{\mathfrak {M}}))}$. Thus, (3.24) yields

3.25 $$\begin{aligned} \frac{\textrm{d}(\eth \phi )}{\textrm{d}s} + \frac{v^2}{\gamma f(s)} \frac{\eth \phi }{\widetilde{\mathfrak {M}}\,D(\widetilde{\mathfrak {M}})}\ge 0. \end{aligned}$$

Using the integrating factor $\exp \left( \frac{v^2}{\gamma } \int _{\widetilde{s}_\nu }^s \frac{\textrm{d}\varrho }{f\,\widetilde{\mathfrak {M}}\,D(\widetilde{\mathfrak {M}})}\right)$ and integrating in $(\widetilde{s}_\nu ,s)$ one has

3.26 $$\begin{aligned} \eth \phi (s) \ge \eth \phi (\widetilde{s}_\nu )\, \exp \left( -\frac{v^2}{\gamma } \int _{\widetilde{s}_\nu }^s \frac{\textrm{d}\varrho }{f(\varrho )\,\widetilde{\mathfrak {M}}(\varrho )\,D(\widetilde{\mathfrak {M}}(\varrho ))}\right) . \end{aligned}$$

Observe from (3.21) and (3.10a) that $\frac{\textrm{d}\widetilde{\mathfrak {M}}}{\textrm{d}s}<0$ for $s\in (\widetilde{s}_\nu ,1)$, which gives

3.27 $$\begin{aligned} -\frac{v^2}{\gamma } \frac{1}{f\,\widetilde{\mathfrak {M}}\,D(\widetilde{\mathfrak {M}})}\overset{(3.10a)}{=} \frac{1}{\widetilde{\mathfrak {M}}(s)-\ell (s)}\frac{\textrm{d}\widetilde{\mathfrak {M}}}{\textrm{d}s}= \tfrac{1}{\left( 1-\frac{\ell (s)}{\widetilde{\mathfrak {M}}(s)}\right) }\frac{1}{\widetilde{\mathfrak {M}}}\frac{\textrm{d}\widetilde{\mathfrak {M}}}{\textrm{d}s} \overset{(3.21)}{\ge }\frac{1}{\nu } \frac{1}{\widetilde{\mathfrak {M}}}\frac{\textrm{d}\widetilde{\mathfrak {M}}}{\textrm{d}s}. \end{aligned}$$

Putting this in (3.26), we have

$$\begin{aligned} \eth \phi (s) \ge \eth \phi (\widetilde{s}_\nu )\, \exp \left( \frac{1}{\nu }\int _{\widetilde{s}_\nu }^s \frac{\textrm{d}\widetilde{\mathfrak {M}}}{\widetilde{\mathfrak {M}}}\right) = \eth \phi (\widetilde{s}_\nu ) \left( \frac{\widetilde{\mathfrak {M}}(s)}{\widetilde{\mathfrak {M}}(\widetilde{s}_\nu )}\right) ^{\frac{1}{\nu }}. \end{aligned}$$

Rearranging the above relation using (3.22) one obtains for a constant $\widetilde{C}^1_\nu >0$ only dependent on $\widetilde{s}_\nu <1$ that

3.28 $$\begin{aligned} \Phi (\widetilde{\mathfrak {M}}) + (\widetilde{C}^1_\nu \widetilde{\mathfrak {M}})^{\frac{1}{\nu }}\le \Phi (\mathfrak {M}), \quad \text { or }\quad \widetilde{C}^1_\nu \widetilde{\mathfrak {M}}\le (\Phi (\mathfrak {M}))^{\nu }. \end{aligned}$$

Recalling (P1) and the definition of $\Phi$ in (3.9), we choose $\nu \in (0,1)$ such that there exists a constant $\widetilde{C}^2_\nu >0$ for which

3.29 $$\begin{aligned} \Phi (m)^{\nu }\le \widetilde{C}^2_\nu \Phi '(m)\overset{(3.9)}{=} \widetilde{C}^2_\nu \, m\,D(m) \text { for all } m\in (0,1). \end{aligned}$$

In a right neighbourhood $[0,\varepsilon ]$ of $m=0$ ($\epsilon >0$), where $D(m)\sim C m^a$ (see (P1)) for some constant $C>0$, we have $\Phi '(m)\sim C m^{a+1}$ and $\Phi (m)\sim \frac{C}{a+2} m^{a+2}$. Hence, for any constant $\nu \in [(1+a)/(2+a), 1)$ there exists $\widetilde{C}^2_\nu >0$ satisfying (3.29) in $[0,\varepsilon ]$. For $m\in (0,1)$, one can take $\widetilde{C}^2_\nu$ to be the minimum of the value of $\widetilde{C}^2_\nu$ for $m\in [0,\varepsilon ]$, and $\min _{m\in (\varepsilon ,1)} \Phi (m)^{\nu } (\Phi '(m))^{-1}>0$. Then, for $s\in (\widetilde{s}_\nu ,1)$ one has

3.30 $$\begin{aligned} \widetilde{\zeta }(s)-\widetilde{\zeta }(\widetilde{s}_\nu )&= \frac{v}{\gamma }\int _{\widetilde{s}_\nu }^s \frac{\textrm{d}\varrho }{f(\varrho )\,\widetilde{\mathfrak {M}}(\varrho )}\overset{(3.28)}{\ge }\frac{v}{\gamma \widetilde{C}^1_\nu }\int _{\widetilde{s}_\nu }^s\frac{\textrm{d}\varrho }{f(\varrho )\,\Phi (\mathfrak {M}(\varrho ))^{\nu }}\nonumber \\&\overset{(3.29)}{\ge }\frac{v}{\gamma \widetilde{C}^1_\nu \widetilde{C}^2_\nu } \int _{\widetilde{s}_\nu }^s \frac{\textrm{d}\varrho }{f(\varrho )\,\mathfrak {M}(\varrho ) D(\mathfrak {M}(\varrho ))}\overset{(3.5b)}{=} \frac{1}{\widetilde{C}^1_\nu \widetilde{C}^2_\nu }\int _{\widetilde{s}_\nu }^s \left( \frac{\textrm{d}\tau }{\textrm{d}S}\right) \textrm{d}S. \end{aligned}$$

The orbit $(M,S)\rightarrow (0,1)$ only as $\tau \rightarrow \infty$ since (0, 1) is an equilibrium point of () and the right hand side of () is locally Lipschitz with respect to *M* and *S*. Hence, the right hand side of (3.30) tends to $+\infty$ as $s\nearrow 1$. This contradicts $\widetilde{\xi }=\widetilde{\zeta }(1)$ being bounded, which concludes the proof of the proposition.

By this point we have found a unique orbit which satisfies half of the boundary conditions in (2.12), i.e. $(M,S)(0)=(0,1)$. For the boundary condition at the other end, i.e. $\xi \rightarrow -\infty$, we investigate how the orbit varies with *v*.

### Ordering of the Orbits with Respect to *v*

**Fig. 4:**
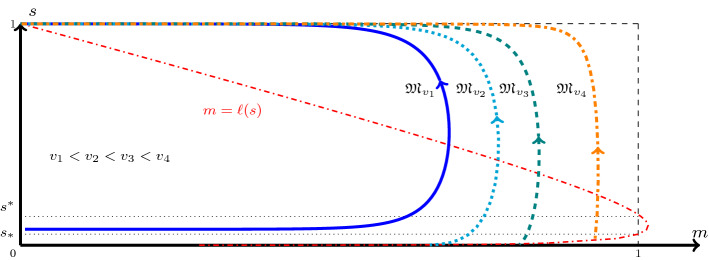
The ordering of orbits as stated in Theorem 3.2 computed for the parameter set provided in [10] (Color figure online)

#### Theorem 3.2

(The ordering of the orbits with respect to *v*) For $v>0$, let the function $\mathfrak {M}_v:[s_{-\infty },1)\rightarrow [0,1]$ denote the $\mathfrak {M}$-mapping introduced in Theorem 3.1 that satisfies $\mathfrak {M}_v(1)=0$. Then, for a fixed $s\in [s_{-\infty },1)$, $\mathfrak {M}_v(s)$ varies continuously with *v*. Moreover,

(i) $\mathfrak {M}_v(s)$ is strictly increasing with *v*.
(ii) $$\mathfrak {M}_v(s)\rightarrow 0$$ as $$v\rightarrow 0$$.
(iii) Let $\mathcal {G}(s)>0$ for all $s\in (s_{-\infty },1)$. Then, $\mathfrak {M}_v(s)\rightarrow 1$ as $v\rightarrow \infty$.

Schematically the ordering of the orbits is shown in Fig. 4.

#### Proof

*(i)* For a fixed $v>0$, let $\mathfrak {M}^\varepsilon _v$ denote the $\mathfrak {M}$-mapping discussed in Lemma 3.2 with $\mathfrak {M}^\varepsilon _v(1)=\varepsilon >0$. Let $0<v_1<v_2$. Observe that

$$\begin{aligned} \frac{\textrm{d}}{\textrm{d}s}\Phi (\mathfrak {M}^\varepsilon _{v_1}(1))=-\frac{v^2_1}{\gamma \, f(1)}\varepsilon > -\frac{v^2_2}{\gamma \, f(1)}\,\varepsilon =\frac{\textrm{d}}{\textrm{d}s}\Phi (\mathfrak {M}^\varepsilon _{v_2}(1)), \end{aligned}$$

implying $\Phi (\mathfrak {M}^\varepsilon _{v_1}(s))< \Phi (\mathfrak {M}^\varepsilon _{v_2}(s))$ in a left neighbourhood of $s=1$. We show below that $\Phi (\mathfrak {M}_{v_1}^{\varepsilon }(s))< \Phi (\mathfrak {M}_{v_2}^{\varepsilon }(s))$ for all $s\in [s_{-\infty },1)$ provided $\mathfrak {M}_{v_2}^{\varepsilon }(s)>0$. To show this, let us assume the contrary, i.e. $\mathfrak {M}_{v_1}^\varepsilon (s_1)=\mathfrak {M}_{v_2}^\varepsilon (s_1)>0$ for some $s_1\in [s_{-\infty },1)$ with $\mathfrak {M}^\varepsilon _{v_1}(s)< \mathfrak {M}^\varepsilon _{v_2}(s)$ for all $s\in (s_1,1)$. Subtracting two versions of (3.10b), we obtain

3.31 $$\begin{aligned} \frac{\textrm{d}}{\textrm{d}s} (\Phi (\mathfrak {M}_{v_1}^\varepsilon (s))-\Phi (\mathfrak {M}_{v_2}^\varepsilon (s)))&=\frac{1}{f(s)}\left[ -\frac{v^2_1}{\gamma }(\mathfrak {M}_{v_1}^\varepsilon -\mathfrak {M}_{v_2}^\varepsilon ) -\dfrac{v_2^2-v_1^2}{\gamma }[\ell (s)-\mathfrak {M}_{v_2}^\varepsilon ] \right] \nonumber \\&=-\frac{v^2_1}{\gamma \, f(s)}(\mathfrak {M}_{v_1}^\varepsilon -\mathfrak {M}_{v_2}^\varepsilon ) -\left( 1-\frac{v_1^2}{v_2^2}\right) \frac{\textrm{d}}{\textrm{d}s} \Phi (\mathfrak {M}_{v_2}^\varepsilon (s)). \end{aligned}$$

Integrating in $(s_1,1)$ we get

$$\begin{aligned} \Phi (\mathfrak {M}_{v_2}^\varepsilon (s_1))-\Phi (\mathfrak {M}_{v_1}^\varepsilon (s_1))= \frac{v^2_1}{\gamma }\int _{s_1}^1(\mathfrak {M}_{v_2}^\varepsilon -\mathfrak {M}_{v_1}^\varepsilon )\frac{\textrm{d}s}{f(s)}+ \left( 1-\frac{v_1^2}{v_2^2}\right) \Phi (\mathfrak {M}_{v_2}^\varepsilon (s_1))>0, \end{aligned}$$

thus contradicting our assumption. Hence, $\Phi (\mathfrak {M}_{v_1}^{\varepsilon }(s))< \Phi (\mathfrak {M}_{v_2}^{\varepsilon }(s))$ for all $s\in [s_{-\infty },1)$ and passing to the limit $\varepsilon \rightarrow 0$ we obtain

3.32 $$\begin{aligned} \Phi (\mathfrak {M}_{v_1}(s))\le \Phi (\mathfrak {M}_{v_2}(s)) \text { for all } s\in [s_{-\infty },1). \end{aligned}$$

To prove that $\mathfrak {M}_{v}$ strictly increases with *v*, we observe integrating (3.10b) in (*s*, 1) that

$$\begin{aligned} \frac{\Phi (\mathfrak {M}_{v_2}(s))}{v_2^2}=\frac{1}{\gamma }\int _s^1 [\mathfrak {M}_{v_2}-\ell ](\varrho )\frac{\textrm{d}\varrho }{f(\varrho )}\overset{(3.32)}{\ge }\frac{1}{\gamma }\int _s^1 [\mathfrak {M}_{v_1}-\ell ](\varrho )\frac{\textrm{d}\varrho }{f(\varrho )}=\frac{\Phi (\mathfrak {M}_{v_1}(s))}{v_1^2}, \end{aligned}$$

which yields the important inequality

3.33 $$\begin{aligned} \Phi (\mathfrak {M}_{v_2}(s))\ge \frac{v_2^2}{v_1^2} \Phi (\mathfrak {M}_{v_1}(s))>\Phi (\mathfrak {M}_{v_1}(s)). \end{aligned}$$

This proves that $\mathfrak {M}_v(s)$ is strictly increasing with respect to *v*.

*(ii)* Integrating (3.10b) in (*s*, 1) we have

$$\begin{aligned} \Phi (\mathfrak {M}_v(s))=\frac{v^2}{\gamma }\int _s^1 (\mathfrak {M}_v(\varrho )-\ell (\varrho ))\frac{\textrm{d}\varrho }{f(\varrho )}< \frac{v^2}{\gamma }\int _s^1 (1-\ell (\varrho ))\frac{\textrm{d}\varrho }{f(\varrho )}\overset{(3.4)}{=}\left( \frac{v}{\gamma }\right) ^2 \mathcal {G}(s). \end{aligned}$$

This proves point (ii).

*(iii)* We prove the statement first in $(s^*,1)$. Recall that $s^*\in (0,1)$ satisfies $\ell (s^*)=1$. Let $S^{\dagger }_v\in (s^*,1)$ be such that $\mathfrak {M}_v(S^{\dagger }_v)=\ell (S^{\dagger }_v)$. The existence of $S^{\dagger }_v$ is guaranteed by Corollary 3.1. Also, point (i) implies that $S^{\dagger }_v$ decreases as *v* increases. Let $S^\dagger = \lim \limits _{v\rightarrow \infty } S^{\dagger }_v$. Then Corollary 3.1 yields again

3.34 $$\begin{aligned} \max _{s\in (s_*,1)}\mathfrak {M}_v(s)=\ell (S^{\dagger }_v), \text { and hence, } \sup _{v>0}\max _{s\in (s_*,1)}\mathfrak {M}_v(s) = \ell (S^{\dagger }). \end{aligned}$$

Let $S^\dagger >s^*$, yielding $\ell (S^{\dagger })<1$. Then using (3.33) one has for some $v_1=v>0$ and $v_2=v^2$ that

$$\begin{aligned} \Phi (\mathfrak {M}_{v_2}(S^{\dagger }_{v_1}))\ge \frac{v_2^2}{v_1^2} \Phi (\mathfrak {M}_{v_1}(S^{\dagger }_{v_1}))= v^2 \Phi (\ell (S^{\dagger }_{v}))\rightarrow \infty , \text { as } v\rightarrow \infty , \end{aligned}$$

which contradicts the upper bound in (3.34) since $\ell (S^\dagger )<1$. Hence, $\lim \limits _{v\rightarrow \infty } S^{\dagger }_v=s^*$.

Now let $s\in (s^*,1)$. Then, there exists $v_1>0$ such that $S^{\dagger }_{v_1}\ge s$. Using (3.33) for $v>v_1$,

3.35 $$\begin{aligned} \Phi (\mathfrak {M}_v(s))\ge \frac{v^2}{v_1^2} \Phi (\mathfrak {M}_{v_1}(s))\ge \frac{v^2}{v_1^2} \Phi (\ell (s))\rightarrow \infty \text { as } v\rightarrow \infty . \end{aligned}$$

Hence, $\mathfrak {M}_v(s)\rightarrow 1$ for all $s\in (s^*,1)$.

Now, we extend the result to $(s_{-\infty },s^*)$. Set $\check{s}_0=s^*$ and let $\check{s}_k\in [s_{-\infty },\check{s}_{k-1}]$, $k\in {\mathbb {N}}$, be recursively defined by the formula

$$\begin{aligned} \int _{\check{s}_k}^{\check{s}_{k-1}} \frac{\ell (\varrho )}{f(\varrho )}\, \textrm{d}\varrho = \int ^{1}_{\check{s}_{k-1}} \frac{1-\ell (\varrho )}{f(\varrho )}\, \textrm{d}\varrho \overset{(2.15)}{=} \gamma ^{-1} \mathcal {G}(\check{s}_{k-1})>0. \end{aligned}$$

If such $\check{s}_k$ does not exist in $[s_{-\infty },\check{s}_{k-1}]$ then we set $\check{s}_k=s_{-\infty }$. Assume that $\lim _{v\rightarrow \infty }\mathfrak {M}_v(s)\rightarrow 1$ holds for all $s\in (\check{s}_{k-1},1)$. This is certainly true for $k=1$. Then we show that it holds for all $s\in (\check{s}_{k},1)$. For all $s\in (\check{s}_k,\check{s}_{k-1})$ one has

3.36 $$\begin{aligned} \Phi (\mathfrak {M}_v(s))&= \frac{v^2}{\gamma } \int _s^1 \frac{1}{f}(\mathfrak {M}_v-\ell )= \frac{v^2}{\gamma } \left[ \int _{\check{s}_{k-1}}^1 \frac{1}{f}(\mathfrak {M}_v-\ell ) + \int ^{\check{s}_{k-1}}_{s} \frac{1}{f} (\mathfrak {M}_v-\ell )\right] \nonumber \\&\ge \frac{v^2}{\gamma } \left[ \int _{\check{s}_{k-1}}^1 \frac{1}{f} (\mathfrak {M}_v-\ell ) - \int ^{\check{s}_{k-1}}_{s} \frac{\ell }{f} \right] . \end{aligned}$$

The integral $\int _{\check{s}_{k-1}}^1 \frac{1}{f}(\mathfrak {M}_v-\ell ) \rightarrow \int _{\check{s}_{k-1}}^1 \frac{1}{f} (1-\ell ) \overset{(3.4)}{=}\gamma ^{-1}\mathcal {G}(\check{s}_{k-1})$ as $v\rightarrow \infty$. Thus $\int _{\check{s}_{k-1}}^1 \frac{1}{f}(\mathfrak {M}_v-\ell ) - \int ^{\check{s}_{k-1}}_{s} \frac{\ell }{f} >0$ for large *v* and $s>\check{s}_k$. Hence, passing $v\rightarrow \infty$ in (3.36) one gets, $\mathfrak {M}_v(s)\rightarrow 1$ for all $s> \check{s}_k$, thus proving the statement.

*Continuity:* Finally, we prove that $\lim \limits _{v\rightarrow v_0} \mathfrak {M}_v(s)=\mathfrak {M}_{v_0}(s)$ for any $v_0>0$. Let us handle the case $v\searrow v_0$ first. From point (i), $\lim \limits _{v\searrow v_0} \mathfrak {M}_v(s)\ge \mathfrak {M}_{v_0}(s)$. Let us assume

$$\begin{aligned} \lim \limits _{v\searrow v_0} \mathfrak {M}_v(s)=:\bar{\mathfrak {M}}_{v_0}(s)> \mathfrak {M}_{v_0}(s) \text { for some } s\in (s_{-\infty },1). \end{aligned}$$

Just as in the proof of Theorem 3.1, $\bar{\mathfrak {M}}_{v_0}$ satisfies () with $v=v_0$. Since $\mathfrak {M}^\varepsilon _{v_0}(s)\searrow \mathfrak {M}_{v_0}(s)$ as $\varepsilon \searrow 0$ ($\mathfrak {M}^{\varepsilon }_{v_0}$ as defined in Lemma 3.2 for $v=v_0$), one can choose $\varepsilon >0$ small enough such that $\mathfrak {M}_{v_0}(s)<\mathfrak {M}_{v_0}^\varepsilon (s)< \bar{\mathfrak {M}}_{v_0}(s)$. Then $\mathfrak {M}^{\varepsilon }_{v_0}$ and $\bar{\mathfrak {M}}_{v_0}$ both satisfy () and

$$\begin{aligned} (\mathfrak {M}^\varepsilon _{v_0}- \bar{\mathfrak {M}}_{v_0})(1)=\varepsilon >0, \text { whereas } (\mathfrak {M}^\varepsilon _{v_0}- \bar{\mathfrak {M}}_{v_0})(s)<0, \end{aligned}$$

implying that they intersect at some intermediate point. Since both $\mathfrak {M}^{\varepsilon }_{v_0}$ and $\bar{\mathfrak {M}}_{v_0}$ correspond to orbits in the phase–plane with the same $v=v_0$, and the orbits cannot intersect in $\{m>0\}$ we have our contradiction.

The proof of the case $v\nearrow v_0$ follows from Proposition 3.1 since $\bar{\mathfrak {M}}_{v_0}=\lim _{v\nearrow v_0} \mathfrak {M}_v< \mathfrak {M}_{v_0}$ would imply that there are two mappings, $\mathfrak {M}_{v_0}$ and $\bar{\mathfrak {M}}_{v_0}$, which satisfy $\mathfrak {M}(1)=0$ with a corresponding $\zeta (1)=0$, thus contradicting Proposition 3.1.

From the proof of Theorem 3.2 (ii), we obtain the following property:

#### Corollary 3.2

(Bounds on $\mathfrak {M}_v$) For $v>0$, let the function $\mathfrak {M}_v:[s_{-\infty },1]\rightarrow [0,1]$ denote the $\mathfrak {M}$-mapping introduced in Theorem 3.1 that satisfies $\mathfrak {M}_v(1)=0$. Then, for all $v_0<v$ one has

$$\begin{aligned} \frac{v^2}{v_0^2}\Phi (\mathfrak {M}_{v_0}(s))\le \Phi (\mathfrak {M}_{v}(s))\le \left( \frac{v}{\gamma }\right) ^2 \mathcal {G}(s). \end{aligned}$$

### Existence/Non-existence of Travelling Waves

#### Proof of Theorem 2.1

Theorem 3.1 and Proposition 3.1 prove the existence of a unique orbit $(M_v,S_v)$ satisfying () and connecting with (0, 1) at $\xi =0$. Corollary 3.1 shows that the orbit crosses the line $m=\ell (s)$ for some $s\in (s^*,1)$ and Lemma 3.1 shows that it enters $\mathfrak {R}=[0,1)\times (s_{-\infty },1]$ through either $\{m=0,\, s\in [s_{-\infty },1]\}$ or through $\{s=s_{-\infty }\}$. Theorem 3.2 proves that the orbit varies continuously with *v*, and for large *v*, the orbit enters through $s=s_{-\infty }$. Hence, it remains to be shown that for small *v*, the orbit enters through $\{m=0, s\in [s_{-\infty },1]\}$. This will prove, by continuity, the existence of $v=\bar{v}>0$ such that the corresponding orbit connects with $(0,s_{-\infty })$ which is the intersection point of the two segments.

We show that for any $\hat{s}\in [s_{-\infty },1)$ there exists a corresponding $\hat{v}>0$ such that

$$\begin{aligned} \mathfrak {M}_{\hat{v}}(\hat{s})=0 \text { for all } v\le \hat{v}. \end{aligned}$$

Assume that no such $\hat{v}>0$ exists. Then $\mathfrak {M}_v(\hat{s})>0$ for all $v>0$. Integrating (3.10b) in $(\hat{s},1)$ one then has

$$\begin{aligned} 0<\Phi (\mathfrak {M}_v(\hat{s}))= \frac{v^2}{\gamma }\int _{\hat{s}}^1 (\mathfrak {M}_v(\varrho )-\ell (\varrho ))\,\frac{\textrm{d}\varrho }{f(\varrho )}. \end{aligned}$$

However, Corollary 3.2 implies that $\int _{\hat{s}}^1 (\mathfrak {M}_v(\varrho )-\ell (\varrho ))\,\frac{\textrm{d}\varrho }{f(\varrho )}<0$ for small enough $v>0$, since $\mathfrak {M}_v(\varrho )\searrow 0$ uniformly as $v\searrow 0$. This is a contradiction to $\Phi (\mathfrak {M}_v(\hat{s}))>0$. Hence, the hypothesized $\hat{v}$ exists. Setting $\hat{s}=s_{-\infty }$ proves Theorem 2.1.

**Fig. 5:**
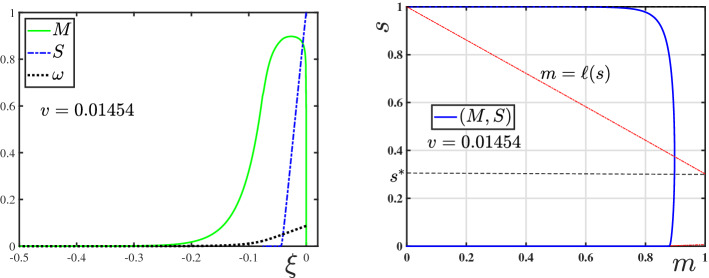
The TW profile for the default parameter set given in Table 1 obtained using Algorithm 5.1. In the (left) plot the graphs of $M(\xi )$, $S(\xi )$ and $\omega (\xi )=\int _{-\infty }^\xi M$ are shown. The (right) plot shows the orbit (*M*, *S*) in the phase–plane (note that $s_{-\infty }\approx 10^{-60}$ in this case). The corresponding wave-speed is $v=0.01454$ (Color figure online)

The profiles of (*M*, *S*) as functions of $\xi \le 0$ are shown in Fig. 5. An interesting feature of this TW is that the profile of *M* has a sharp front at $\xi =0$, whereas, it has a diffused tail at the rear. The TW is in fact a travelling pulse since it connects an equilibrium state with $M=0$ to another equilibrium state with $M=0$, in contrast to TWs for nonlinear diffusion problems with Fisher type source terms, see [3, 4, 11].

#### Remark 3.2

(Consistency of the TW solution) Let (*M*, *S*) be the TW solution described in Theorem 2.1 and let $\mathfrak {M}$ denote its $\mathfrak {M}$-mapping. Then $\ell (s)/\mathfrak {M}\rightarrow 0$ as $s\nearrow 1$ from (3.20), and consequently recalling the TW Assumption 2.1 one has

3.37 $$\begin{aligned} {\left\{ \begin{array}{ll}-D(M)\partial _x M=-D(M)\tfrac{\textrm{d}}{\textrm{d}\xi }M=v(M-\ell (S))\rightarrow 0,\\ -\tfrac{D(M)}{M}\partial _x M=-\tfrac{D(M)}{M}\tfrac{\textrm{d}}{\textrm{d}\xi }M=v\left( 1-\tfrac{\ell (S)}{M}\right) \rightarrow v , \end{array}\right. } \quad \text { as } x-vt=\xi \nearrow 0. \end{aligned}$$

Here, the term $-D(M)\partial _x M$ represents the flux and the term $-\tfrac{D(M)}{M}\partial _x M$ is commonly referred to as the **particle speed** in the literature of the porous medium equation, see [29, Chapter 19]. The fact that the flux vanishes at $\xi =0$ and the particle velocity is *v* implies that the TW is physically consistent with the model (1.1).

#### Remark 3.3

(Regularity of the sharp front) From the limits in (3.37), one can also deduce the regularity of $M(\xi )$ as $\xi \nearrow 0$. Taking the expression (1.3a) for *D*, one has in the limit $\xi \nearrow 0$, observing that $M(\xi )\searrow 0$, that $\delta \,M^{a-1}\,\tfrac{\textrm{d}}{\textrm{d}\xi }M\sim -v$, or

3.38 $$\begin{aligned} M(\xi )\sim \tfrac{a\,v}{\delta }|\xi |^{\frac{1}{a}}, \text { for } \xi \text { in a left neighbourhood of } 0. \end{aligned}$$

Consequently, a sharp front exists at $\xi =0$, however, the TW profile still remains Hölder continuous. The Hölder continuity of solutions of the scalar biofilm model, with diffusion coefficient *D* as in (1.3), has been shown in [15].

Finally, the techniques developed in this section also allow us to give necessary conditions for existence of the TWs.

#### Proposition 3.2

(Non-existence of travelling wave solutions) Let (2.13) be satisfied and $\mathcal {G}(\bar{s})<0$ for some $\bar{s}\in (s_{-\infty },1)$. Then there exists no TW orbit (*M*, *S*) satisfying both () and the boundary conditions (2.12). Similarly, if $\lambda \ge 1$ then no TW exists.

#### Proof

For $\mathcal {G}(\bar{s})<0$, assume that such a solution exists for some $v>0$. Then, integrating () in $(\bar{s},1)$ one has

$$\begin{aligned} 0< \frac{\gamma }{v^2}\Phi (\mathfrak {M}_v(\bar{s}))=\int _{\bar{s}}^1 (\mathfrak {M}_v(\varrho )-\ell (\varrho ))\,\frac{\textrm{d}\varrho }{f(\varrho )}< \int _{\bar{s}}^1 (1-\ell )\,\frac{\textrm{d}\varrho }{f(\varrho )}= \gamma ^{-1}\mathcal {G}(\bar{s})<0, \end{aligned}$$

which is a contradiction. For the case $\lambda \ge 1$, we have from (3.2) that $\ell '(s)\overset{(2.8)}{=}\frac{1}{\gamma f(s)}[f(s)-\lambda ]\overset{\mathrm{(P2)}}{\le }0$ and hence no $s_{-\infty }\in (0,1)$ satisfying (2.10) exists, thus giving the result.

## Linear Stability of the Travelling Waves in Two Space Dimensions

In this section, we analyse the linear stability of the TW solutions in two space dimensions. It was observed numerically in [10, 16] that the TWs are stable with respect to random perturbations, and that solutions resulting from arbitrary initial data converge to a TW solution in a long rectangular domain. We were unable to prove a full nonlinear stability result for the problem. However, in this section, we follow the methodology presented in [28] and use asymptotic expansions to prove linear $L^1$-stability of the TWs under certain (suitable) assumptions. The stability of the TWs is further investigated numerically in Sect. 5.

### Asymptotic Expansion and Linearization

Let $(M^{\langle {0} \rangle },S^{\langle {0} \rangle })$ denote the TWs introduced in Theorem 2.1. For a given $L>0$, we consider the infinite strip $\Omega :={\mathbb {R}}\times (0,L)$ as the domain. Let $(x,y)\in \Omega$ denote the spatial coordinates. Then, we consider the two dimensional version of (1.1) in $\Omega$:

4.1a $$\begin{aligned}&\partial _t M=\partial _{x} [D(M)\,\partial _x M] + \partial _{y}[D(M)\,\partial _y M] + \left( f(S)-\lambda \right) M, \end{aligned}$$

4.1b $$\begin{aligned}&\partial _t S= -\gamma \,f(S)\,M. \end{aligned}$$

We consider the following boundary conditions for (4.1):

4.2a $$\begin{aligned}&M(\pm \infty ,y,t)=0, \;\; (D(M)\,\partial _x M)(\pm \infty ,y,t)=0&\text { for all } y\in (0,L) \text { and } t>0, \end{aligned}$$

4.2b $$\begin{aligned}&(D(M)\,\partial _y M)(x,0,t)=(D(M)\,\partial _y M)(x,L,t)=0&\text { for all } x\in {\mathbb {R}}\text { and } t>0. \end{aligned}$$

The boundary condition (4.2a) generalizes (1.2) to two dimensions, whereas, (4.2b) introduces homogeneous Neumann conditions for the lateral boundaries. As initial condition we choose for an arbitrarily small $\varepsilon >0$,

4.3a $$\begin{aligned} {\left\{ \begin{array}{ll} M(x,y,0)&{}=M^{\langle {0} \rangle }(x) + \varepsilon \sum _{n=0}^\infty M^{\langle {1} \rangle }_{n,0}(x) \cos \left( \Lambda _n\, y\right) ,\\ S(x,y,0)&{}=S^{\langle {0} \rangle }(x) + \varepsilon \sum _{n=0}^\infty S^{\langle {1} \rangle }_{n,0}(x) \cos \left( \Lambda _n\, y\right) , \end{array}\right. } \text { where } \Lambda _n:=\tfrac{2\pi n}{L}, \end{aligned}$$

and the functions $M^{\langle {1} \rangle }_{n,0},\; S^{\langle {1} \rangle }_{n,0}\in L^1({\mathbb {R}}^-)$ are bounded, smooth, and satisfy

4.3b $$\begin{aligned} M^{\langle {1} \rangle }_{n,0}(0)= S^{\langle {1} \rangle }_{n,0}(0)=0, \text { and } \lim \limits _{x\searrow -\infty } M^{\langle {1} \rangle }_{n,0}(x)=\lim \limits _{x\searrow -\infty } S^{\langle {1} \rangle }_{n,0}(x)=0 \text { for all } n\in {\mathbb {N}}_0. \end{aligned}$$

#### Remark 4.1

(Generality of the initial condition) The initial condition (4.3) can be generalized to include also $\sin (\Lambda _n y)$ components which then covers all smooth initial conditions having periodic boundaries at $y\in \{0,\,L\}$ (by Fourier series expansion). The main result of this section (Theorem 4.1) remains unchanged provided that $\sin (\Lambda _n y)$ contributions are added to (4.8). For simplicity, we have used only the $\cos (\Lambda _n y)$ components here. Observe that the initial condition (4.3) is consistent with the zero Neumann conditions in (4.2b).

We assume that the solution of (1.1) can be described in this case by the asymptotic expansion

4.4 $$\begin{aligned} {\left\{ \begin{array}{ll} M(x,y,t)=M^{\langle {0} \rangle }(x-vt) + \varepsilon M^{\langle {1} \rangle }(x,y,t)+\dots ,\\ S(x,y,t)=S^{\langle {0} \rangle }(x-vt) + \varepsilon S^{\langle {1} \rangle }(x,y,t)+\dots , \end{array}\right. } \end{aligned}$$

where $v>0$ represents the wave speed, and $M^{\langle {1} \rangle }_{n}$, $S^{\langle {1} \rangle }_n$ are differentiable functions such that the boundary conditions (4.2) and initial conditions (4.3) are satisfied. Then by inserting the expansion (4.4) in (4.1) and equating the $\varepsilon$-order terms one obtains the system

4.5a $$\begin{aligned} \partial _t M^{\langle {1} \rangle }&= \partial _{xx}\left( D \left( M^{\langle {0} \rangle }\right) M^{\langle {1} \rangle }\right) + \partial _{yy}\left( D \left( M^{\langle {0} \rangle }\right) M^{\langle {1} \rangle }\right) \nonumber \\&\quad + \left( f \left( S^{\langle {0} \rangle }\right) -\lambda \right) M^{\langle {1} \rangle } + f' \left( S^{\langle {0} \rangle }\right) M^{\langle {0} \rangle } S^{\langle {1} \rangle }, \end{aligned}$$

4.5b $$\begin{aligned} \partial _t S^{\langle {1} \rangle }&=-\gamma \left[ f \left( S^{\langle {0} \rangle }\right) M^{\langle {1} \rangle } + f' \left( S^{\langle {0} \rangle }\right) M^{\langle {0} \rangle } S^{\langle {1} \rangle }\right] . \end{aligned}$$

Since the TW coordinate, $\xi =x-vt$, is a more natural space-coordinate compared to *x* to analyse this problem, we use the coordinate transform $(x,y,t)\mapsto (\xi ,y',t')$, where

4.6 $$\begin{aligned} \xi =x-vt,\; y'=y \text { and } t'=t, \text { giving } \partial _t=\partial _{t'} -v\partial _{\xi },\; \partial _x=\partial _{\xi } \text { and } \partial _{y'}=\partial _y. \end{aligned}$$

System () is modified accordingly. For simplicity, referring to $(y',t')$ as (*y*, *t*), we then have the following problem

4.7a $$\begin{aligned} \partial _t M^{\langle {1} \rangle }-v\,\partial _{\xi } M^{\langle {1} \rangle }&= \partial _{\xi \xi }\left( D\left( M^{\langle {0} \rangle }\right) M^{\langle {1} \rangle }\right) + \partial _{yy}\left( D \left( M^{\langle {0} \rangle }\right) M^{\langle {1} \rangle }\right) \nonumber \\&\quad + \left( f \left( S^{\langle {0} \rangle }\right) -\lambda \right) M^{\langle {1} \rangle } + f' \left( S^{\langle {0} \rangle }\right) M^{\langle {0} \rangle } S^{\langle {1} \rangle }, \end{aligned}$$

4.7b $$\begin{aligned} \partial _t S^{\langle {1} \rangle }-v\,\partial _{\xi } S^{\langle {1} \rangle }&=-\gamma \left[ f \left( S^{\langle {0} \rangle }\right) M^{\langle {1} \rangle } + f' \left( S^{\langle {0} \rangle }\right) M^{\langle {0} \rangle } S^{\langle {1} \rangle }\right] . \end{aligned}$$

Due to the form of the initial condition prescribed, we look for $M^{\langle {1} \rangle }$ and $S^{\langle {1} \rangle }$ of the following form

4.8 $$\begin{aligned} {\left\{ \begin{array}{ll} M^{\langle {1} \rangle }(x,y,t)&{}=\sum \limits _{n=0}^\infty M^{\langle {1} \rangle }_{n}(\xi ,t) \cos \left( \Lambda _n\, y\right) ,\\ S^{\langle {1} \rangle }(x,y,t)&{}=\sum \limits _{n=0}^\infty S^{\langle {1} \rangle }_{n}(\xi ,t) \cos \left( \Lambda _n\,y\right) , \end{array}\right. } \text { with } \Lambda _n=\tfrac{2\pi n}{L}. \end{aligned}$$

Substituting (4.8) in (4.7), observing that $(M^{\langle {0} \rangle },S^{\langle {0} \rangle })$ only depends on $\xi$, and equating the $\cos \left( \Lambda _n y\right)$ terms, we have for each $n\in {\mathbb {N}}_0$ the system

4.9a $$\begin{aligned} \partial _t M^{\langle {1} \rangle }_n-v\,\partial _{\xi } M^{\langle {1} \rangle }_n&= \partial _{\xi \xi }\left( D\left( M^{\langle {0} \rangle }\right) M^{\langle {1} \rangle }_n \right) - \Lambda _n^2 M^{\langle {1} \rangle }_n\nonumber \\&\quad + \left( f\left( S^{\langle {0} \rangle }\right) -\lambda \right) M^{\langle {1} \rangle }_n + f' \left( S^{\langle {0} \rangle }\right) M^{\langle {0} \rangle } S^{\langle {1} \rangle }_n, \end{aligned}$$

4.9b $$\begin{aligned} \partial _t S^{\langle {1} \rangle }_n-v\,\partial _{\xi } S^{\langle {1} \rangle }_n&=-\gamma \left[ f \left( S^{\langle {0} \rangle }\right) M^{\langle {1} \rangle }_n + f' \left( S^{\langle {0} \rangle }\right) M^{\langle {0} \rangle } S^{\langle {1} \rangle }_n \right] . \end{aligned}$$

In order to satisfy the boundary and initial conditions (4.2)–(4.3) for each $n\in {\mathbb {N}}_0$ one must have

4.10a $$\begin{aligned} M^{\langle {1} \rangle }_n(0,t)&= S^{\langle {1} \rangle }_n(0,t) =0,&\text { for all } t>0, \end{aligned}$$

4.10b $$\begin{aligned} \lim \limits _{\xi \searrow -\infty } M^{\langle {1} \rangle }_n(\xi ,t)&=\lim \limits _{\xi \searrow -\infty } S^{\langle {1} \rangle }_n(\xi ,t)= 0,&\text { for all } t>0, \end{aligned}$$

4.10c $$\begin{aligned} M^{\langle {1} \rangle }_n(\xi ,0)&=M^{\langle {1} \rangle }_{n,0}(\xi ),\quad S^{\langle {1} \rangle }_n(\xi ,0)=S^{\langle {1} \rangle }_{n,0}(\xi )&\text { for all } \xi <0. \end{aligned}$$

#### Remark 4.2

(Choice of boundary conditions ()) The boundary conditions at $\xi =0$ prescribed in () imply that $\partial _\xi ( D(M^{\langle {0} \rangle }) M^{\langle {1} \rangle }_n)=D(M^{\langle {0} \rangle }) \partial _\xi M^{\langle {1} \rangle }_n + \partial _\xi D(M^{\langle {0} \rangle }) M^{\langle {1} \rangle }_n= 0$ at $\xi =0$ since $\partial _{\xi } D(M^{\langle {0} \rangle })$ is bounded. Thus, the flux is zero at $\xi =0$ which ensures that $(M^{\langle {1} \rangle }_n(t),S^{\langle {1} \rangle }_n(t))$, as a solution to (), can be extended to $(0,\infty )$ by setting

$$\begin{aligned} M^{\langle {1} \rangle }_n=S^{\langle {1} \rangle }_n=0, \text { for all } \xi>0 \text { and } t>0. \end{aligned}$$

Hence, () is satisfied for all $\xi \in {\mathbb {R}}$ and $t>0$. Finally, the boundary conditions $M^{\langle {1} \rangle }_n=S^{\langle {1} \rangle }_n=0$ at $\xi =-\infty$ is consistent with the initial conditions in (4.3) and make it possible to have absolutely integrable solutions $(M^{\langle {1} \rangle }_n,S^{\langle {1} \rangle }_n)$.

### Stability in $L^1$-Norm

#### Theorem 4.1

(Stability of the travelling waves) Let $(M^{\langle {0} \rangle },S^{\langle {0} \rangle }):{\mathbb {R}}^-\rightarrow [0,1]^2$ be a travelling wave solution satisfying ()–(2.12) with a given wave-speed $v>0$. Assume that for all $n\in {\mathbb {N}}_0$, a continuously differentiable and absolutely integrable solution $(M^{\langle {1} \rangle }_n,S^{\langle {1} \rangle }_n):{\mathbb {R}}^-\times [0,\infty ) \rightarrow {\mathbb {R}}^2$ exists of the problem () which satisfies the initial and boundary conditions (). Then, for any given $t>0$, one has

4.11 $$\begin{aligned}&\int _{{\mathbb {R}}^-}\left[ |M^{\langle {1} \rangle }_n(t)| + \tfrac{1}{\gamma }|S^{\langle {1} \rangle }_n(t)|\right] + \left( \lambda + \Lambda _n^2\right) \int _0^t \int _{{\mathbb {R}}^-} |M^{\langle {1} \rangle }_n| \nonumber \\&\le \int _{{\mathbb {R}}^-}\left[ |M^{\langle {1} \rangle }_{n,0}| + \tfrac{1}{\gamma }|S^{\langle {1} \rangle }_{n,0}|\right] + \int _0^t \int _{\{M^{\langle {1} \rangle }_n\cdot S^{\langle {1} \rangle }_n\le 0\}} [2 f(S^{\langle {0} \rangle })|M^{\langle {1} \rangle }_n|-f'(S^{\langle {0} \rangle })M^{\langle {0} \rangle }| S^{\langle {1} \rangle }_n|]. \end{aligned}$$

Consequently, if $\Lambda _n^2> 2 f(1)-\lambda >0$, then $\int _0^\infty \int _{{\mathbb {R}}^-} |M^{\langle {1} \rangle }_n|<\infty$, and

4.12 $$\begin{aligned}&\int _{{\mathbb {R}}^-}\left[ |M^{\langle {1} \rangle }_n(t)| + \tfrac{1}{\gamma }|S^{\langle {1} \rangle }_n(t)|\right] \text { is strictly decreasing with respect to } t>0. \end{aligned}$$

#### Remark 4.3

(Stability of the travelling waves) $\text {If }\; L< {2\pi }/{\sqrt{2 f(1)-\lambda }}$, then (4.12) holds for all $n\in {\mathbb {N}},$ thus proving stability of the TWs in two dimensions. However, for $n=0$ one has $\Lambda _n=0$, and thus, the stability of the TW is not guaranteed. In practice, this means that perturbations to the TW that have fast transverse variations decay. The case $n=0$ represents no perturbation in the transverse direction but only in the direction of the TW. Unfortunately, only conditional stability can be proven for this case using our analysis. Increased stability due to transverse variations, similar to Theorem 4.1, has been studied earlier, for example in [28]. Using numerical simulations we show in Sect. 5.1.1 that the TWs are also stable in terms of large longitudinal perturbations.

#### Proof

The proof uses the well-known $L^1$-contraction principle. We reproduce a formal version here for the sake of brevity.

Let $\textrm{sign}_\varepsilon :{\mathbb {R}}\rightarrow [-1,1]$ denote a regularised version of the signum function for $\varepsilon >0$ with $\mathcal {U}_\varepsilon$ as its primitive. More precisely,

4.13 $$\begin{aligned} \textrm{sign}_\varepsilon (u):={\left\{ \begin{array}{ll} 1 &{}\text { if } u>\varepsilon ,\\ u/\varepsilon &{}\text { if } |u|\le \varepsilon ,\\ -1 &{}\text { if } u<-\varepsilon ,\\ \end{array}\right. } \text { and }\; \mathcal {U}_\varepsilon (u)=\int _0^u \textrm{sign}_\varepsilon . \end{aligned}$$

Note the following properties of these functions for future use

4.14a $$\begin{aligned}&|u\, {\textrm{sign}_\varepsilon }'(u)|{\left\{ \begin{array}{ll} <1 \text { for } |u|\le \varepsilon ,\\ =0 \text { for } |u|>\varepsilon ,\\ \end{array}\right. } \text { and }\; \mathcal {U}_\varepsilon (u)\ge 0 \text { with equality only for } u=0, \end{aligned}$$

4.14b $$\begin{aligned}&\textrm{sign}_\varepsilon (u)\rightarrow \textrm{sign}(u), \; u \,\textrm{sign}_\varepsilon (u)\rightarrow |u|, \text { and } \mathcal {U}_\varepsilon (u)\rightarrow |u| \text { pointwise as } \varepsilon \searrow 0. \end{aligned}$$

We use $\textrm{sign}_\varepsilon (M^{\langle {1} \rangle }_n)$ as a test function in (4.9a). Multiplying (4.9a) by $\textrm{sign}_\varepsilon (M^{\langle {1} \rangle }_n)$ and integrating in ${\mathbb {R}}^-$ one has term by term

4.15a $$\begin{aligned}&\int _{{\mathbb {R}}^-} \textrm{sign}_\varepsilon \left( M^{\langle {1} \rangle }_n \right) \,\partial _t M^{\langle {1} \rangle }_n=\partial _t \left( \int _{{\mathbb {R}}^-} \mathcal {U}_\varepsilon \left( M^{\langle {1} \rangle }_n \right) \right) \overset{(4.14b)}{\rightarrow }\partial _t \left( \int _{{\mathbb {R}}^-} |M^{\langle {1} \rangle }_n|\right) \text { as } \varepsilon \searrow 0, \end{aligned}$$

4.15b $$\begin{aligned} -v&\int _{{\mathbb {R}}^-} \textrm{sign}_\varepsilon \left( M^{\langle {1} \rangle }_n \right) \,\partial _\xi M^{\langle {1} \rangle }_n = -v\int _{{\mathbb {R}}^-} \partial _\xi \,\mathcal {U}_\varepsilon \left( M^{\langle {1} \rangle }_n \right) = v \left[ \mathcal {U}_\varepsilon \left( M^{\langle {1} \rangle }_n(-\infty ,t)\right) - \mathcal {U}_\varepsilon (0)\right] \overset{(4.10)}{=} 0. \end{aligned}$$

From the second order term, one has using integration by parts, and the boundary conditions in () that

4.15c $$\begin{aligned} \int _{{\mathbb {R}}^-} \textrm{sign}_\varepsilon \left( M^{\langle {1} \rangle }_n \right) \, \partial _{\xi \xi }\left( D \left( M^{\langle {0} \rangle }\right) M^{\langle {1} \rangle }_n \right) =&\, - \int _{{\mathbb {R}}^-} \partial _{\xi }\left( \textrm{sign}_\varepsilon \left( M^{\langle {1} \rangle }_n \right) \right) \partial _{\xi }(D \left( M^{\langle {0} \rangle }\right) M^{\langle {1} \rangle }_n)\nonumber \\ =&\,- \int _{{\mathbb {R}}^-} {\textrm{sign}_\varepsilon }' \left( M^{\langle {1} \rangle }_n \right) D \left( M^{\langle {0} \rangle }\right) |\partial _{\xi } M^{\langle {1} \rangle }_n|^2 \nonumber \\ {}&\,- \int _{{\mathbb {R}}^-} {\textrm{sign}_\varepsilon }' \left( M^{\langle {1} \rangle }_n \right) \,M^{\langle {1} \rangle }_n\, \partial _\xi \left( D \left( M^{\langle {0} \rangle }\right) \right) \partial _{\xi } M^{\langle {1} \rangle }_n\nonumber \\ \overset{(4.14a)}{\le }&\, \int _{\{|M^{\langle {1} \rangle }_n|<\varepsilon \}} |\partial _\xi D \left( M^{\langle {0} \rangle }\right) \,\partial _{\xi } M^{\langle {1} \rangle }_n|\rightarrow 0 \qquad \text { as } \varepsilon \searrow 0. \end{aligned}$$

Finally, for the source terms, one has as $\varepsilon \rightarrow 0$,

4.15d $$\begin{aligned}&\int _{{\mathbb {R}}^-} \textrm{sign}_\varepsilon \left( M^{\langle {1} \rangle }_n \right) \,\left[ \left( f \left( S^{\langle {0} \rangle }\right) -\lambda -\Lambda _n^2 \right) M^{\langle {1} \rangle }_n + f' \left( S^{\langle {0} \rangle }\right) M^{\langle {0} \rangle } S^{\langle {1} \rangle }_n \right] \nonumber \\&\overset{(4.14b)}{\rightarrow }- \left( \lambda +\Lambda _n^2 \right) \int _{{\mathbb {R}}^-} |M^{\langle {1} \rangle }_n| + \int _{{\mathbb {R}}^-} \textrm{sign}\left( M^{\langle {1} \rangle }_n \right) \,\left[ f\left( S^{\langle {0} \rangle }\right) M^{\langle {1} \rangle }_n + f'\left( S^{\langle {0} \rangle }\right) M^{\langle {0} \rangle } S^{\langle {1} \rangle }_n \right] . \end{aligned}$$

Similarly, multiplying (4.9b) by $\textrm{sign}_\varepsilon (S^{\langle {1} \rangle }_n)$, integrating in ${\mathbb {R}}^-$ and following the steps of () one has

4.16 $$\begin{aligned} \partial _t \left( \int _{{\mathbb {R}}^-} |S^{\langle {1} \rangle }_n|\right) \le -\gamma \int _{{\mathbb {R}}^-} \textrm{sign}(S^{\langle {1} \rangle }_n)\,\left[ f \left( S^{\langle {0} \rangle }\right) M^{\langle {1} \rangle }_n + f' \left( S^{\langle {0} \rangle }\right) M^{\langle {0} \rangle } S^{\langle {1} \rangle }_n \right] . \end{aligned}$$

Adding ()–(4.16) one thus obtains

4.17 $$\begin{aligned}&\partial _t \left( \int _{{\mathbb {R}}^-}\left[ |M^{\langle {1} \rangle }_n| + \tfrac{1}{\gamma }|S^{\langle {1} \rangle }_n|\right] \right) + \left( \lambda + \Lambda _n^2\right) \int _{{\mathbb {R}}^-} |M^{\langle {1} \rangle }_n|\nonumber \\&\le \int _{{\mathbb {R}}^-} \left( \textrm{sign}\left( M^{\langle {1} \rangle }_n \right) -\textrm{sign}\left( S^{\langle {1} \rangle }_n \right) \right) \,\left[ f \left( S^{\langle {0} \rangle }\right) M^{\langle {1} \rangle }_n + f'\left( S^{\langle {0} \rangle }\right) M^{\langle {0} \rangle } S^{\langle {1} \rangle }_n \right] . \end{aligned}$$

Note that if both $M^{\langle {1} \rangle }_n,\, S^{\langle {1} \rangle }_n>0$ or $M^{\langle {1} \rangle }_n,\, S^{\langle {1} \rangle }_n<0$ then the right hand side vanishes. On the other hand, if $M^{\langle {1} \rangle }_n> 0$ and $S^{\langle {1} \rangle }_n<0$ then $\textrm{sign}\left( M^{\langle {1} \rangle }_n \right) -\textrm{sign}(S^{\langle {1} \rangle }_n)=2$ and $f(S^{\langle {0} \rangle }) M^{\langle {1} \rangle }_n + f'(S^{\langle {0} \rangle }) M^{\langle {0} \rangle } S^{\langle {1} \rangle }_n= f(S^{\langle {0} \rangle }) |M^{\langle {1} \rangle }_n|- f'(S^{\langle {0} \rangle }) M^{\langle {0} \rangle } |S^{\langle {1} \rangle }_n|$. Hence, the integrand on the right hand side becomes

$$\begin{aligned} 2 (f(S^{\langle {0} \rangle }) |M^{\langle {1} \rangle }_n|- f'(S^{\langle {0} \rangle }) M^{\langle {0} \rangle } |S^{\langle {1} \rangle }_n|)< 2f(S^{\langle {0} \rangle }) |M^{\langle {1} \rangle }_n|- f'(S^{\langle {0} \rangle }) M^{\langle {0} \rangle } |S^{\langle {1} \rangle }_n|. \end{aligned}$$

By symmetry, we have the same inequality when $M^{\langle {1} \rangle }_n< 0$ and $S^{\langle {1} \rangle }_n>0$. Including the trivial cases of $M^{\langle {1} \rangle }_n= 0$ and/or $S^{\langle {1} \rangle }_n=0$ which in both cases yield

$$\begin{aligned}{} & {} (\textrm{sign}\left( M^{\langle {1} \rangle }_n \right) -\textrm{sign}(S^{\langle {1} \rangle }_n))\,[f(S^{\langle {0} \rangle }) M^{\langle {1} \rangle }_n + f'(S^{\langle {0} \rangle }) M^{\langle {0} \rangle } S^{\langle {1} \rangle }_n] \\{} & {} \quad \le 2f(S^{\langle {0} \rangle }) |M^{\langle {1} \rangle }_n|- f'(S^{\langle {0} \rangle }) M^{\langle {0} \rangle } |S^{\langle {1} \rangle }_n|, \end{aligned}$$

we have (4.11) by integrating (4.17) in time.

Since $f(S^{\langle {0} \rangle })< f(1)$ in ${\mathbb {R}}^-$, we have $\lambda +\Lambda _n^2>2f(S^{\langle {0} \rangle })$ in (4.12). This implies that there exists a constant $c_1>0$ such that for any $0<t_1<t_2$,

$$\begin{aligned} \int _{{\mathbb {R}}^-}\left[ |M^{\langle {1} \rangle }_n(t_2)| + \tfrac{1}{\gamma }|S^{\langle {1} \rangle }_n(t_2)|\right] + c_1\int _{t_1}^{t_2} \int _{{\mathbb {R}}^-} |M^{\langle {1} \rangle }_n| \le \int _{{\mathbb {R}}^-}\left[ |M^{\langle {1} \rangle }_{n}(t_1)| + \tfrac{1}{\gamma }|S^{\langle {1} \rangle }_{n}(t_1)|\right] . \end{aligned}$$

Hence, $\int _{{\mathbb {R}}^-}\left[ |M^{\langle {1} \rangle }_n(t)| + \tfrac{1}{\gamma }|S^{\langle {1} \rangle }_n(t)|\right]$ is a decreasing function having a limit, and $\int _0^t \int _{{\mathbb {R}}^-} |M^{\langle {1} \rangle }_n|$ is bounded uniformly for all $t>0$.

## Numerical Results

In this section, we verify the analytical predictions of Sects. 3 and 4 numerically by computing solutions of the PDE systems (1.1) and (4.1). It is shown that for an arbitrary initial condition, the PDE solutions indeed converge to a profile moving with constant speed both in one and two space dimensions, which further shows numerically the stability of the TWs. The TWs are also obtained directly by solving () and finding the correct wave-speed by a bisection algorithm. The TWs produced by the PDE simulations and the bisection algorithm are shown to coincide. The algorithm is then used to further investigate the parametric dependence of the TW profiles and wave-speed for a greater range of parameters. Finally, a numerical continuation approach is considered which enables us to study the limiting cases for which the assumptions in Theorem 2.1 are satisfied.

### PDE Simulations

Here, we solve the PDE systems (1.1) (one space dimension) and (4.1) (two space dimensions) with expressions (1.3) for $D,\,f$, on finite rectangular domains using homogeneous Neumann boundary conditions. The PDEs are solved using the standard two-point flux approximation finite volume method. The solution is approximated at the centres of each grid cell. The diffusion across the cell interfaces are approximated via arithmetic averaging as described in [9]. For time integration we use the trapezoidal method with a uniform time-step size of $\Delta t$. The large system of arithmetic equations generated by this approach is solved via a fixed point iteration scheme described in [16]. The default set of simulation parameters is given in Table 1. These parameters will be used throughout this section unless stated otherwise.

**Table 1:** Default model parameters (with names used in the context of cellulolytic biofilm models) used for the simulations of (1.1), (4.1) and ()

Model parameter	Symbol	Value	Reference
Motility coefficient in (1.3)	$\delta$	$10^{-6}$	[10]
Diffusion exponent 1 in (1.3)	*a*	4.0	[10]
Diffusion exponent 2 in (1.3)	*b*	4.0	[10]
Half saturation concentration in (1.3)	$\kappa$	0.01	[10]
Maximum consumption rate	$\gamma$	0.4	[10]
Cell-loss rate	$\lambda$	0.42	[10]

The functions *D* and *f* are as in (1.3). The diffusion exponent *a* controls steepness of the front, and *b* controls how close to the singular value ($m=1$) the biomass concentration *M* can get. All values in the table are dimensionless

#### One Dimensional Results: Transience and Stability

In this case, our spatial domain is (0, *H*) for $H>0$. The default initial conditions for the system are for $h\in (0,1)$ and $d\in (0,H)$,

5.1 $$\begin{aligned} M(x,0)={\left\{ \begin{array}{ll} h\left( 1-\tfrac{x^4}{d^4}\right) &{} \text {if } x\le d,\\ 0 &{} \text {otherwise}, \end{array}\right. } \quad S(x,0)\equiv 1. \end{aligned}$$

The numerical parameters used for the simulation are

5.2 $$\begin{aligned} H=1,\;\; \Delta x=2^{-N}, \;\; \Delta t=10^{-2}\,(2^9\Delta x)^2,\;\; d=\tfrac{5}{127},\;\; h=0.1. \end{aligned}$$

In the above, *N* is an integer between 9 and 16. A grid independence study is done in Appendix 1 and based on the result the default value of $N=14$ is chosen.

**Fig. 6:**
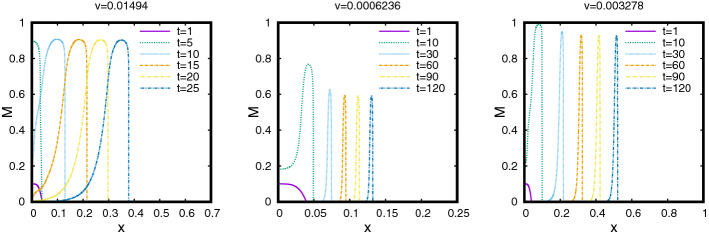
Transient behaviour of the *M*-profiles obtained from the PDE simulations. The parameters are taken from Table 1 except in (left) $\lambda =0.42$, (center) $\lambda =0.60$, and (right) $a=b=2$ (Color figure online)

**Fig. 7:**
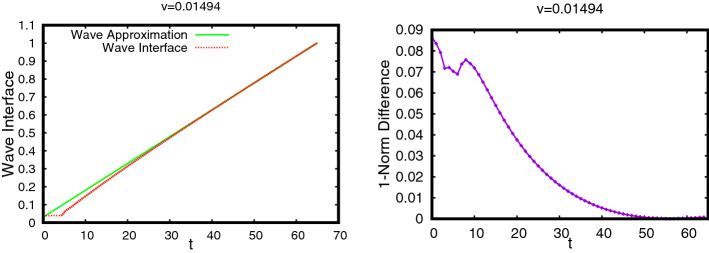
(left) Wave-speed calculation for the default parameters in Table 1 using linear fitting. The resulting wave-speed estimated is $v=0.01494$. (right) The $L^1$-difference $\int _0^H |M(t)-M(64)|$ plotted against time *t* for the same simulation (Color figure online)

Observe that the initial condition (5.1) is arbitrary and has no relation to the TWs. Nevertheless, the numerical solutions develop into profiles that move with a constant speed. This is shown in Fig. 6 using three sets of parameters. To estimate the wave-speed of the developed profile, we calculate the interface of the biomass wave by taking the largest *x*-coordinate such that $M(x,t)>10^{-2}$. We use $10^{-2}$ as an approximation of 0 to avoid any numerical noise generated due to the degeneracy. Once we have the wave interface, we can estimate the wave speed by fitting a linear function through the data points corresponding to the wave interface. This is done using the built-in function $\textsf {fit}$ from GNUPLOT which gives the wave-speed. Figure 7 (left) illustrates the process for the parameter set in Table 1. The (right) plot, on the other hand, shows the difference of *M*(*t*) and the developed profile at $t=64$ in the $L^1$-norm as a function of time *t*. It clearly shows the convergence of the *M*-profiles emanating from the initial condition (5.1) to a TW moving with speed $v=0.01494$. Unless otherwise stated, all the PDE-simulations are verified to permit a TW solution hereinafter.

#### Two Dimensional Results: Transience and Stability

For the two dimensional results our spatial domain is the rectangle $[0,H]\times [0,L]$. The initial condition prescribed is

5.3 $$\begin{aligned} M(x,y,0)={\left\{ \begin{array}{ll} h\left( 1-\tfrac{x^4}{d^4}\right) \left( 1+ \tfrac{1}{5}\cos \left( \frac{2\pi y}{L}\right) \right) &{} \text {if } x\le d,\\ 0 &{} \text {otherwise}, \end{array}\right. } \quad S(x,y,0)\equiv 1. \end{aligned}$$

The numerical parameters for this case are

5.4 $$\begin{aligned} H=2,\;\; L=1,\;\; \Delta x=2^{-9}, \;\; \Delta t=0.01,\;\; d=\tfrac{5}{127},\;\; h=0.1. \end{aligned}$$

**Fig. 8:**
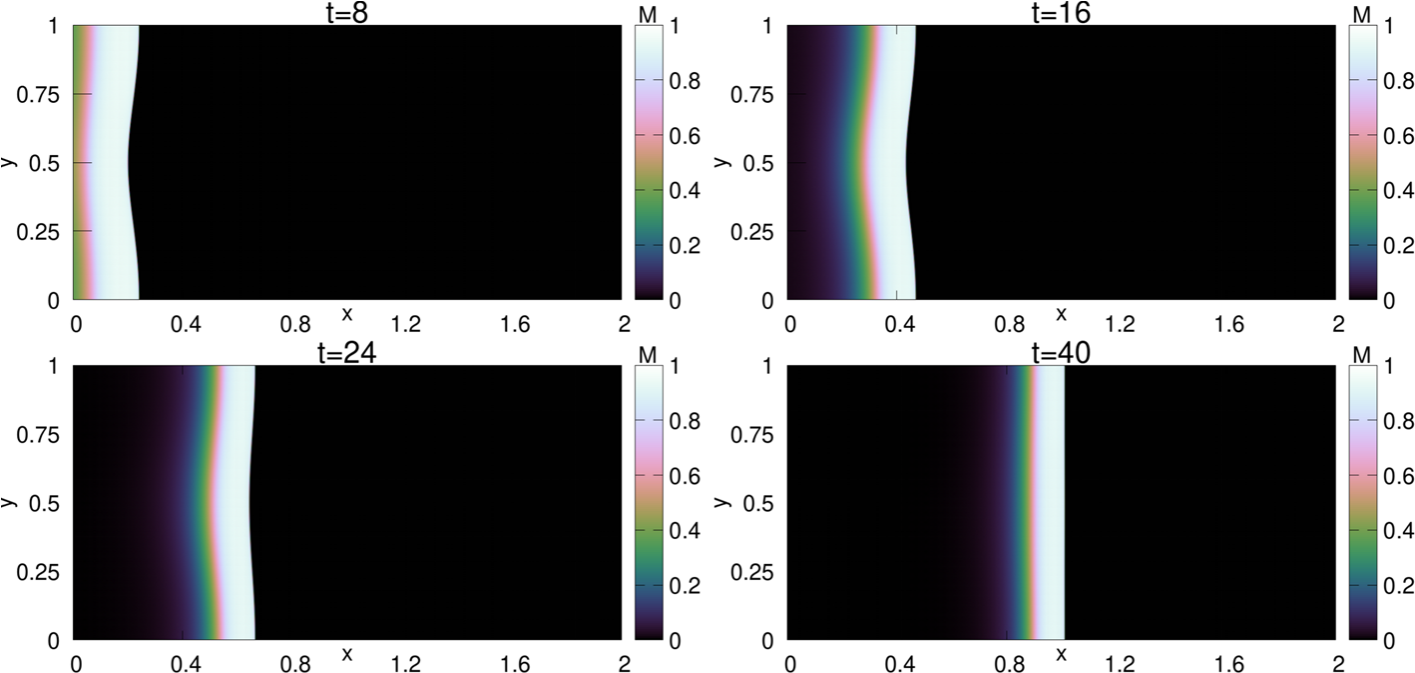
The transient behaviour of the numerical solution of (4.1) subjected to the initial condition (5.3). The parameter values are taken from Table 1 (Color figure online)

**Fig. 9:**
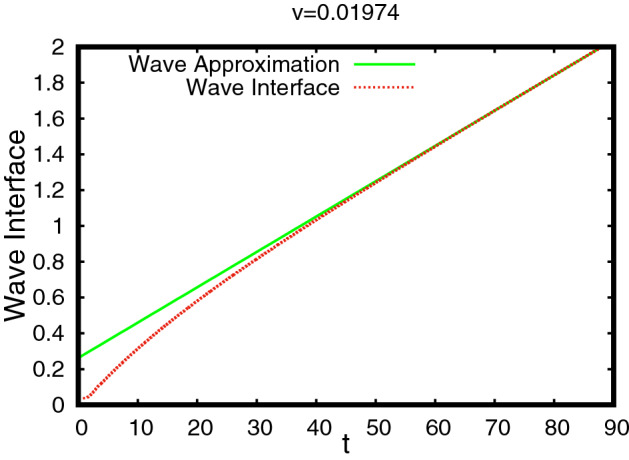
Wave-speed estimation for the two dimensional simulation presented in Fig. 8 (Color figure online)

As before, the initial condition in this case is arbitrary and deviates largely from any TW profile both in terms of its *x* and *y* variations. As such, it does not satisfy the restrictions imposed in Sect. 4 and Theorem 4.1 for proving linear stability. Nevertheless, Fig. 8 shows that the numerical solution still develops slowly into a planar front that moves with a constant speed. This speed, estimated in Fig. 9, is very close to the wave-speed computed at the same level of discretization, i.e., $\Delta x=2^{-9}$, for the one dimensional case, see Table 2 of Appendix 1.

### ODE Simulations

Upon verifying that the solutions indeed converge to TW solutions, we devise a more direct and faster method to compute the TW profiles.

#### TW Algorithm

To directly find the TW profile and the corresponding wave-speed $v>0$ for a given set of parameters, we use a semi-analytic approach that utilizes the monotonicity property described in Theorem 3.2 with respect to $v>0$. We solve the dynamical system () for a given $v>0$, except we solve the negation of the vector field, i.e.

5.5a $$\begin{aligned}&\tfrac{\textrm{d}}{\textrm{d}\tau }M^{v}= -v\,[\ell (S^v)- M^v], \end{aligned}$$

5.5b $$\begin{aligned}&\tfrac{\textrm{d}}{\textrm{d}\tau }S^v= -\tfrac{\gamma }{v} {f(S^v) M^v D(M^v)}, \end{aligned}$$

with *D* and *f* as in (1.3). The above system is solved numerically for $\tau >0$ using the 4^th^-order Runge–Kutta method [26] and the following initial condition is used for the computation:

5.6 $$\begin{aligned} (M^v,S^v)(0)=(\varepsilon ,1),\;&\text { for } \varepsilon =10^{-3}. \end{aligned}$$

This avoids the problem of starting from the degenerate equilibrium point (0, 1) and gives a close approximation to the TW profile as indicated by Theorem 3.1.

Then, the **bisection method** is used to determine the wave-speed for which $(M^v,S^v)$ connects to the other equilibrium point $(0,s_{-\infty })$. Let $\bar{v}>0$ be large enough so that $(M^{\bar{v}},S^{\bar{v}})$ exits the region $\mathfrak {R}:=[0,1)\times [s_{-\infty },1]$ through the line $\{s=s_{-\infty }\}$. The existence of such $\bar{v}>0$ is ensured by Theorem 3.2. Similarly, let $\underline{v}\in (0,\bar{v})$ be such that $(M^{\underline{v}},S^{\underline{v}})$ exits $\mathfrak {R}$ through the line $\{m=0\}$. Then we use the following algorithm to determine the wave-speed $v>0$:

##### Algorithm 5.1

(Bisection iteration to determine the travelling wave)

1. Set $v=\frac{1}{2}|\bar{v}+\underline{v}|$. Solve $(M^v,S^v)$ satisfying () and (5.6) numerically.
2. If $(M^v,S^v)$ exits $\mathfrak {R}$ through $s=s_{-\infty }$, then set $\bar{v}=v$. Else, set $\underline{v}=v$.
3. If $|\bar{v}-\underline{v}|<10^{-4}\,v$ then stop; otherwise go to Step 1.

For the default parameter set in Table 1, Algorithm 5.1 is over 1000 times faster than the PDE computation under the same computational set-up.

#### Validation of the TW Algorithm Using the PDE Scheme

**Fig. 10:**
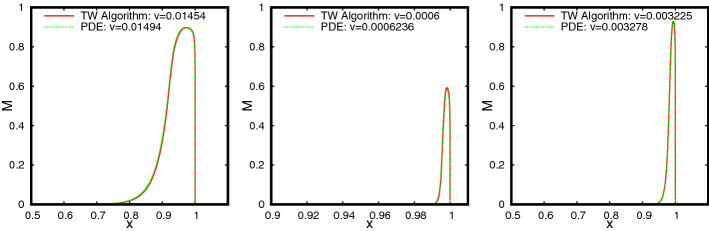
The comparison of the *M*-profiles obtained by using Algorithm 5.1 with the PDE simulations. The parameters are taken from Table 1 except in (left) $\lambda =0.42$, (center) $\lambda =0.60$, and (right) $a=b=2$ (Color figure online)

We compare the profiles obtained from Algorithm 5.1 with the profiles of the full PDE simulations. The same three test cases are chosen as in Fig. 6. The results are shown in Fig. 10. We consider the agreement to be excellent with the wave-speed varying only by $2.72\%$ in the default case of Table 1 for $N=14$ in (5.2), and the profiles are barely distinguishable from each other for the two methods.

#### Parametric Study in the Limiting Cases

Figure 10 shows us that when $\lambda$ changes from 0.42 to 0.6, the wave-speed and the perceived width of the TW become about 20 times smaller. Hence, PDE simulations become impractical both for large $\lambda$ values, due to very small mesh-sizes required, and for small $\lambda$ values, due to the increase in the required domain size. Similar problems occur for the coefficients $a,\,b$ (see Fig. 10) and the parameters $\gamma$ and $\kappa$. Since Algorithm 5.1 is much faster compared to the PDE computations, these cases can be better explored using the ODE simulations.

**Fig. 11:**
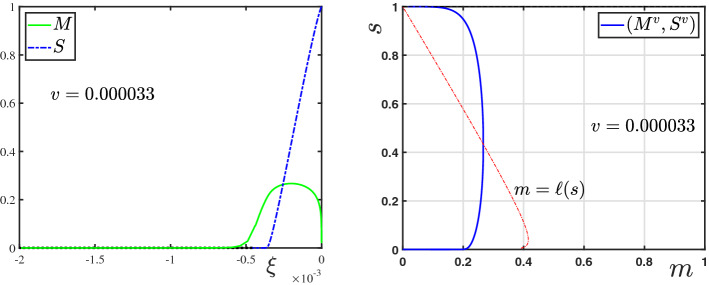
(left) The plots of $M^v$ and $S^v$ vs. $\xi =x-vt$ computed using Algorithm 5.1 for $\lambda =0.8$. (right) The orbit $(M^v,S^v)$ in the *m*–*s* phase–plane (Color figure online)

An interesting case-study is the investigation of the conditions provided in Theorem 2.1 for the existence of TWs. They are, Condition 1: $\mathcal {G}(s)>0$ for all $s\in (s_{-\infty },1)$, which is also a necessary condition due to Proposition 3.2; and Condition 2: (2.13) is satisfied. For $\gamma$ and $\kappa$ given in Table 1, Condition 1 is satisfied if $\lambda \ge 0.26$ whereas Condition 2 is satisfied if $\lambda \le 0.56$. Here we are interested in exploring the limits of $\lambda$ for which the TWs exist. We already saw from Figs. 6 and 10 that a TW solution exists for $\lambda =0.6$, thus indicating that Condition 2 is not a necessary condition. Using the TW algorithm, we also find TW solutions up to $\lambda =0.8$. The profile is much narrower in this case and has a minuscule $v=0.000033$, see Fig. 11 (left). However, we were unable to find any TW solutions for $\lambda \ge 0.9$ which is still smaller than the absolute limit of $\lambda =1$ for which it is guaranteed that TW solutions do not exist, see Proposition 3.2. Theorem 2.1 does not guarantee the existence of TW solutions in these cases since the nullcline $m=\ell (s)$ does not intersect the line $\{m=1\}$, see Fig. 11 (right).

**Fig. 12:**
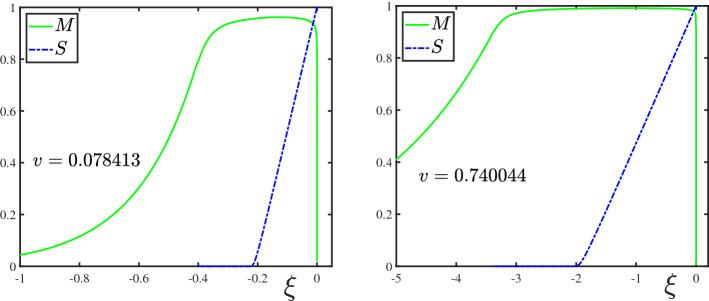
The plots of $M^v$ and $S^v$ vs. $\xi =x-vt$ computed using Algorithm 5.1. For the (left) plot $\lambda =0.38$, and the (right) plot $\lambda =0.36$ (Color figure online)

On the other hand, if $\lambda$ is chosen smaller than $\lambda =0.42$, then we see a rapid increase in wave-speed and a widening of the profile, see Fig. 12. In fact, varying between $\lambda =0.38$ and $\lambda =0.36$ the wave-speed and the profile width increase ten fold. As such, the computational time required for the algorithm to converge increases exponentially, and we were unable to obtain TW solutions for values of $\lambda$ smaller than 0.36. However, the trend in behaviour when varying $\lambda$ is evident from the simulations.

### Numerical Continuation Method

Another approach to study parameter regimes for which the system (3.5) has a connecting orbit is to use numerical continuation techniques [5]. The approach is to find a connecting orbit on the center unstable manifold of the rest state $(0,s_{-\infty })$ to the strong stable manifold of the rest state (0, 1).

Following [5], we set this up as a two point boundary value problem:

5.7a $$\begin{aligned}&\tfrac{\textrm{d}}{\textrm{d}\tau }M= v\,T\, [\ell (S)- M], \quad \text{ for }\,\tau \in (0,1), \end{aligned}$$

5.7b $$\begin{aligned}&\tfrac{\textrm{d}}{\textrm{d}\tau }S= \tfrac{\gamma }{v}\,T\, \,f(S)\, M D(M), \quad \text{ for }\,\tau \in (0,1) ,\end{aligned}$$

5.7c $$\begin{aligned}&(M(0),S(0))= (\varepsilon _0, s_{\varepsilon _0}), \end{aligned}$$

5.7d $$\begin{aligned}&(M(1),S(1)= (\varepsilon _1,1), \end{aligned}$$

where $s_{\varepsilon _0}$ is the smallest root of the equation

$$\begin{aligned} \ell (s)=\varepsilon _0, \end{aligned}$$

i.e., $\ell (s_{\varepsilon _0}) = \varepsilon _0$. Note that (5.7d) is a point on the linearized strong stable manifold for the rest state (0, 1). The center unstable manifold of the rest state $(0,s_{-\infty })$ is well approximated by $(\ell (s),s)$ for *s* near $s_{-\infty }$ since the homological equation for the center manifold is given by

$$\begin{aligned} M D(M)\, \dfrac{\textrm{d}M}{\textrm{d}S} = \frac{v^2}{\gamma f(S)}\,[\ell (S)- M], \end{aligned}$$

and $M D(M) ={ \mathcal O}(M^{1+a})$ for *M* near zero. This is why we use the boundary condition (5.7c). The parameter *T* is the time of travel and is taken to be very large in order to approximate the heteroclinic orbit. An integral condition (see, e.g. [5, 6]) is added to (5.7) to facilitate adaptive mesh selection when computing a branch of solutions. We use AUTO-07P [7] for these computations.

**Fig. 13:**
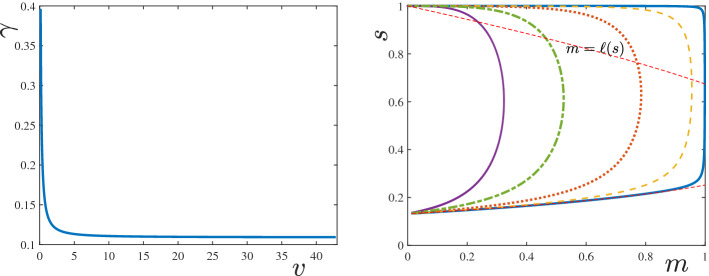
The results for the numerical continuation method. The (left) plot is the bifurcation diagram of $\gamma$ versus the wave speed *v*. In the (right) plot are the corresponding solutions in the phase–plane (Color figure online)

To further illustrate the conclusions of Theorem 2.1, and to investigate the limiting cases where the condition $\mathcal {G}(s)>0$ for all $s\in (s_{-\infty },1)$ is violated, we compute the heteroclinic connections using the parameter values $a=b=2$, $\kappa =1$, and $\lambda = 0.3$. With these values of $\kappa$ and $\lambda$, we find numerically that $s_{-\infty }\approx 0.1319$ and $\mathcal {G}(s)>0$ for all $s\in (s_{-\infty },1)$ provided $\gamma > \approx 0.1093$. Hence, TWs cannot exist for $\gamma$ below this threshold. This is exactly what is observed numerically from Fig. 13 (left) where the wave-speed *v* is plotted against corresponding $\gamma$ values. The horizontal asymptote shows that no solution is possible below a certain threshold of $\gamma$ close to the predicted value. In the (right) figure we see how the orbits vary in the phase–plane when $\gamma$ is decreased, tending towards the line $\{m=1\}$ uniformly. This is similar to what was observed in the case when $\lambda$ was lowered, see Fig. 12.

**Fig. 14:**
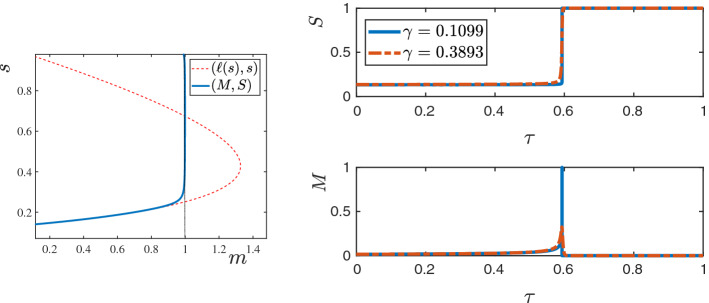
(left) Plot of one solution (blue) in the plase–plane along with the nullcline $m=\ell (s)$ (red). Here $\gamma =0.1099$ and $v=16.227$. (right) Plot of two different solutions as functions of $\tau$, one for $\gamma =0.3893$ (red) and one for $\gamma = 0.1099$ (blue) (Color figure online)

In Fig. 14 (left) we see a representative solution in the phase–plane along with the nullcline, $m=\ell (s)$. The effect of using the phase-condition as described in [6] is illustrated in Fig. 14 (right) where solutions at two different parameter values are depicted.

## Interpretation of the Results

The analysis in Sect. 3 shows that TWs for the system (1.1) exist as expected from the numerical experiments in [10] and observations on cellulolytic biofilms made in [30]. Moreover, we predicted theoretically and verified numerically that they exist for a wide range of parameters. The resulting TWs are found to be stable even for large perturbations (see Figs. 6 and 8) which agrees with the numerical observations in [10, 16]. In the context of biofilm growth, the TWs describe the formation of crater like structures (inverted colonies), i.e. the invasion and degradation of the undisturbed cellulosic environment by microbes. At the leading edge of this invading front is a microbially active layer that consumes the substrate. In the wake of this layer, decay terms dominate over growth terms, and thus, this region is dominated by the dynamics of how fast substrates degrade and how fast bacteria decay if growth cannot be sustained. This complex interplay gives the TW its distinct shape.

Our analysis also suggests that the TWs do not exist if for example, either the cell-loss rate $\lambda \in (0,1)$ is too small or too large (Proposition 3.2). From the numerical experiments in Sect. 5.2, we can infer the reason behind the non-existence of TWs. We see from Fig. 12 that as $\lambda$ decreases, the TW profile becomes wider very quickly, and the biofilm concentration *M* approaches a value close to 1 in a large interval. This is expected since small $\lambda$ implies less decay of *M*, and for very small $\lambda$ we expect that the biofilm concentration would grow monotonically with time and reach 1 in every point of the domain, implying that a TW solution cannot exist. On the other hand, for $\lambda$ large, the profiles become narrower and their amplitudes decrease as seen from Fig. 11. Hence, one expects that for $\lambda$ large enough, the initial biofilm profile would decay to 0 monotonically with time.

The variation of the wave-speed can also be explained through these observations. If $\lambda$ is small, then the profile is wider. Hence, the bacteria consume the substrate faster, which results in a higher wave-speed. For $\lambda$ large the effect is reversed.

Similarly, the effects of the consumption rate $\gamma >0$ on the existence, profile-width and wave-speed of the TW can be explained, see Fig. 13. Higher values of $\gamma$ result in a faster consumption of the substrate, which leads to a decrease in the production of biomass. Hence, the effects of increasing $\gamma$ are analogous to the effects of increasing $\lambda$.

Lastly, we remark that our approach can also be applied to PDE–ODE systems with a porous media type diffusion coefficient (i.e. $D(M)=M^a$), although boundedness of $M\in [0,1)$ cannot be expected in this case. Furthermore, we expect that the results in this paper can be extended to study PDE–ODE systems with multiple substrates, see the system in [12] for instance. Due to the structure of the TW, as a pulse with sharp front and diffusive tail travelling at a constant speed, systems of the form (1.1) can likely be used to model several other biological and physical processes with immobile substrates, such as tumor growth, fungal growth, and the spreading of wildfire, and plant disease.

## Figures and Tables

**Fig. 15:**
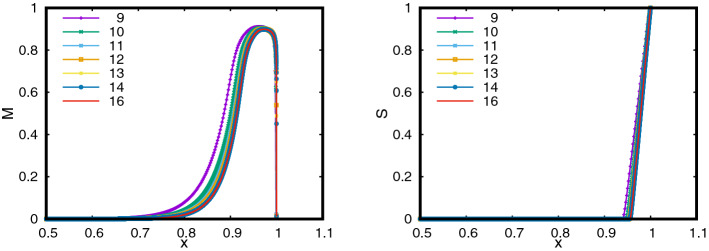
Wave profiles of the various grid refinement simulations. The simulation parameters are given in Table 1 (Color figure online)

## Data Availability

All data generated or analysed during this study are included in this article. The numerical method for the PDE simulations is proposed in [16]. The matlab code for the ODE simulation is uploaded in https://github.com/koondax/TW_ODEsim.git. AUTO-07P [7] (https://github.com/auto-07p/auto-07p) is used for the numerical continuation method. Further simulation data can be provided by the corresponding author on reasonable request. Other models used in this paper are from articles that are publicly available, and they have been properly referenced.

## Appendix A. Grid Independence Study for the PDE Simulations

In this section, we study how the mesh-size $\Delta x$ affects the TW solution obtained numerically from solving the PDE. The focus is to investigate the convergence of the wave-speed and profile as $\Delta x\rightarrow 0$. Let $v_N$ denote the wave-speed, numerically measured using the scheme described in Sect. 5.1.1, for the $2^N$-grid simulation. We quantify the relative differences between the wave-speeds across grid resolutions via the ratios

A.1 $$\begin{aligned} \textrm{ReD}_N=\frac{|v_N-v_{16}|}{|v_{16}|}, \text { and } \textrm{GRR}_N=\frac{\textrm{ReD}_{N-1}}{\textrm{ReD}_N}. \end{aligned}$$

Here, the quantity $\textrm{GRR}_N$ stands for the grid refinement ratio which gives a quantitative comparison between the successive relative differences. The wave-speeds for each simulation along with the relative difference calculations are given in Table 2.

**Table 2:** Wave-speeds calculated from the various grid resolutions along with the relative difference calculations ReD_N_ and GRR_N_ from (A.1). The wave speed for the $2^{16}$ grid simulation is $v_{16}=0.014868$

N	Grid size	Wave-speed $v_N$	ReD_N_	GRR_N_
9	512	0.019450	0.308242	-
10	1024	0.017100	0.150125	2.05
11	2048	0.015909	0.070031	2.14
12	4096	0.015335	0.031434	2.23
13	8192	0.015064	0.013201	2.38
14	16384	0.014944	0.005165	2.56

It is seen from Table 2 that the wave-speeds decrease as the grid is refined and relative differences tend to zero. These observations indicate that we have convergence with respect to the wave-speed. Figure 15 shows the convergence of the *M* and *S* profiles as the grid is made finer. A narrower overall wave profile and a steeper wave front is observed with finer grid resolutions. This is due to less numerical diffusion at smaller discretization levels.

The convergence of both the wave-speed and the wave profile guarantees that the TWs can indeed be reproduced using the numerical scheme described in Sect. 5.1. Given the results in Table 2 and considerations on simulation runtime, we use a $2^{14}$-grid in our simulations and a time step of $\Delta t=10^{-2}(2^9(1/2^{14}))^2$ as presented in (5.2).
